# Sertoli cells have a functional NALP3 inflammasome that can modulate autophagy and cytokine production

**DOI:** 10.1038/srep18896

**Published:** 2016-01-08

**Authors:** Soren Hayrabedyan, Krassimira Todorova, Asma Jabeen, Gergana Metodieva, Stavri Toshkov, Metodi V. Metodiev, Milcho Mincheff, Nelson Fernández

**Affiliations:** 1 Institute of Biology and Immunology of Reproduction, Laboratory of Reproductive Omics Technologies, Bulgarian Academy of Sciences, Sofia, Bulgaria; 2 School of Biological Sciences, Wivenhoe Park, University of Essex, Colchester, UK; 3 Cellular and Gene Therapy Ward, National Specialized Haematology Hospital, Sofia, Bulgaria

## Abstract

Sertoli cells, can function as non-professional tolerogenic antigen-presenting cells, and sustain the blood-testis barrier formed by their tight junctions. The NOD-like receptor family members and the NALP3 inflammasome play a key role in pro-inflammatory innate immunity signalling pathways. Limited data exist on NOD1 and NOD2 expression in human and mouse Sertoli cells. Currently, there is no data on inflammasome expression or function in Sertoli cells. We found that in primary pre-pubertal Sertoli cells and in adult Sertoli line, TLR4\NOD1 and NOD2 crosstalk converged in NFκB activation and elicited a NALP3 activation, leading to *de novo* synthesis and inflammasome priming. This led to caspase-1 activation and IL-1β secretion. We demonstrated this process was controlled by mechanisms linked to autophagy. NOD1 promoted pro-IL-1β restriction and autophagosome maturation arrest, while NOD2 promoted caspase-1 activation, IL-1β secretion and autophagy maturation. NALP3 modulated NOD1 and pro-IL-1β expression, while NOD2 inversely promoted IL-1β. This study is proof of concept that Sertoli cells, upon specific stimulation, could participate in male infertility pathogenesis via inflammatory cytokine induction.

Sertoli cells form part of a seminiferous tubule and harbour the spermatogonia. Sertoli cells form tight junctions (TJs) that sustain the blood-testis barrier, which in turn excludes foreign pathogens from the lumen of a seminiferous tubule and shields germ-line auto-antigens from immunocompetent cells^1^,^2^. Innate immune recognition is likely intrinsic in this role, and a number of studies have investigated whether rat, mice, or human Sertoli cells express Toll-like receptor (TLR) family members on their cell surfaces^3^,^4^, and intracellular nucleotide-binding oligomerization domain receptors, or NOD-like receptors (NLRs)^5^,^6^. Unlike TLR, NLRs are primarily intracellular cytoplasmic sensors of pathogen-associated molecular patterns as well as of endogenous products of tissue injury, termed danger-associated molecular patterns^7^. The two most prominent NLRs, nucleotide-binding oligomerization domain containing 1 (NOD1) and 2 (NOD2), are chiefly activated through recognition of specific muropeptide motifs that are present in bacterial peptidoglycan (PG). NOD1 detects the presence of L-Ala-γ-D-Glu-m-diaminopimelic acid (m-DAP), an amino acid characteristic of most Gram-negative and some Gram-positive bacteria. NOD2 detects and directly binds muramyl dipeptide (MDP), a motif that is present in PGs of both Gram-positive and Gram-negative bacteria^8^,^9^. Upon specific ligand binding to either NOD1 or NOD2, the receptors undergo a conformational change, oligomerize, and interact with adapter proteins to trigger a downstream signal.

In bone marrow-derived macrophages (BMDM) and monocytes, oligomeric NOD-like receptors, such as NACHT leucine-rich-repeat protein (NALP) 1, NALP3 and intracellular IL-1β-converting enzyme protease-activating factor, act as stress sensors and promote the assembly of inflammasomes. These sensory multiprotein complexes activate caspase-1, which in turn cleaves pro-interleukin (IL)-1β and results in secretion of the mature cytokine^10^,^11^. It has also been demonstrated that the caspase-1 inflammasome is responsible for UV-induced secretion of IL-1β from human keratinocytes^12^, thus suggesting that pro-IL-1β maturation by the inflammasome is not restricted to just professional-antigen presenting cells.

Currently, NOD1 expression has been confirmed in rat Sertoli cells^3^. No data exist whether inflammasome scaffolds like NALP1 or NLR family, pyrin domain containing 3 (NALP3 or NLRP3) are expressed in murine Sertoli cells, and if there are any functional consequences in regard to Caspase-1 activation and pro-inflammatory cytokine production. Since most uropathogenic bacteria express both LPS and either iE-DAP or eventually MDP, we hypothesized that an operational inflammasome in Sertoli cells might be implicated in a pathogenic feedback mechanism, wherein Sertoli cells, upon immune challenge, would be able to secrete inflammatory cytokines IL-1β or IL-18, thus potentially inaugurating autoimmune-based male infertility. Caspase-1 activation could also directly contribute to NOD-receptors mediated cell death, following an inflammation related form of programmed cell death termed pyroptosis, accompanied with cytokine production, cell swelling and cell burst^13^.

Sertoli cells constantly phagocytize degenerating germ cells and residual bodies during spermiation, and they have been shown to be capable of processing and displaying antigens^14^, a process tightly connected to autophagy^15^. However, the mechanisms underlying this regulation are still poorly understood. Additionally, autophagy-defective macrophages, depleted of either microtubule-associated protein 1 light chain-3 (LC3) or beclin1, displayed enhanced inflammasome-dependent release of IL-1β and IL-18 that was NALP3 mediated^16^.

The innate recognition receptor TLR4 has been considered as an environmental sensor for autophagy-enhancing colocalization of autophagosomes and mycobacteria^17^. The intracellular bacterial sensors NOD1 and NOD2 emerged recently as key candidates linking bacterial sensing and formation of autophagosomes around invasive bacteria^18^. These findings raise the question if NLR and eventually NALP3 activation in Sertoli cells would be accompanied by any autophagy signalling changes.

In the study reported here, we sought to investigate if Sertoli cells express a fully functional NOD-receptor/NALP inflammasome system, utilizing a macrophage-like two-step model of activation with ability to secrete IL-1β. We also investigated if the LPS and iE-DAP responding TLR4 and NOD1 receptors and the NALP3 inflammasome scaffold molecule are interacting with mature autophagosomes, in regard to autophagy regulated IL-1β processing.

## Results

### NOD1 and NOD2 cross-talk in Sertoli cells in NALP3 dependent manner after challenge with iE-DAP and MDP specific ligands

**Figure 1:**
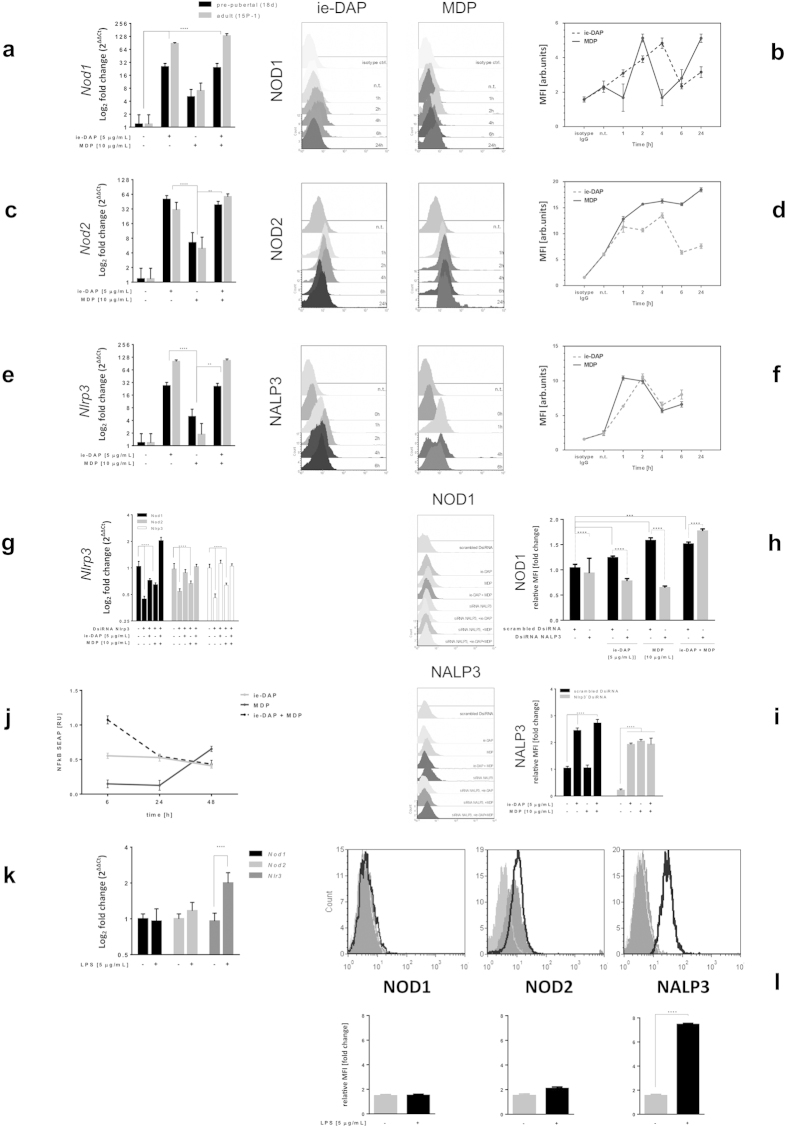
NOD1 and NOD2 cross-talk in Sertoli cells in NALP3 dependent manner after challenge with iE-DAP and MDP specific ligands. (**a,c,e,k**) Quantitative PCR assessment of mRNA transcripts encoding mouse full-length Nod1, Nod2 and Nlrp3 in primary and adult Sertoli cells after challenge (24 h) with iE-DAP, MDP and LPS; (**g**) Nlrp3 silencing (72 h) and challenge (24 h). Data are expressed as log_2_ fold change of non-treated controls compared to ligand challenged ones; **(b, d, f)** Intracellular flow cytometry assessment of protein abundance of NOD1, NOD2 and NALP3 in 15 P-1 cells subject to challenge with iE-DAP and MDP for time points: 1, 2, 4, 6, 24 h; (**h,i**) Nlrp3 silencing (72 h) and challenge (24 h) with iE-DAP, MDP and iE-DAP with MDP; (**l**) LPS challenge (24 h). For each individual experiment data is represented as overlaid histograms of event counts vs. log fluorescence. Protein expression is assessed using the MFI in line graph or bar charts. Data are representative of means ± SD. One representative experiment out of three independent experiments is shown. (**j**) NFκB activation dynamics were followed using stable pNIFTY2-SEAP reporter harbouring 15 P-1 cell line challenged iE-DAP and MDP for 6, 24 and 48 h. The secretory alkaline phosphatase released upon binding to the NFκB response element cassette was measured through cell media collection and colour reading at 405 nm; Error bars indicate SD. Data are representative of three independent experiments with three technical replicates either using primary Sertoli cells of two isolations (n = 80) or the adult Sertoli cell line 15 P-1. ****P < 0.0001; **P < 0.005.

Challenge with iE-DAP or MDP specific ligands for 24 hours significantly increased both NOD1 and NOD2 mRNA and protein expression in primary pre-pubertal and adult 15P-1 Sertoli cells when compared to the non-treated control (Fig. 1a–d, Supplementary Figs 1–4). Similar results were found towards NALP3 mRNA and protein levels (Fig. 1e,f). NOD1 specific iE-DAP ligand induced not only *Nod1*/NOD1, but also *Nod2*/NOD2 in both pre-pubertal and adult Sertoli cells and vice versa, NOD2 specific MDP ligand induced *Nod1*/NOD1 as well. There was an age-related differential gene upregulation, as iE-DAP induced stronger *Nod1* and *Nlrp3* mRNA, while MDP induced weaker *Nod2* and *Nlrp3* mRNA upregulation in adult cells compared to pre-pubertal ones (Fig. 1a–f). Primary pre-pubertal Sertoli cells responded to FSH with an increased NOD1, NOD2 and NALP3 expression, suggesting an improved responsiveness related to pubertal phase, when FSH is physiologically elevated (Supplementary Fig. 5). Since NLR receptor stimulation resulted in cross-talk upregulation of their respective genes after 24 hours challenge, we further investigated the time-dependent dynamics of NOD1, NOD2 and NALP3 expression upon specific ligand challenge for 1, 2, 4, 6 and 24 hours (Fig. 1b,d,f). NOD1 stimulation by iE-DAP produced an early peak in the amount of NOD1 and NOD2 protein, followed by a decrease, provided the stimulation continued for 24 hours. In contrast, MDP stimulation caused a NOD2 plateau opposed by a NOD1 bi-phasic response, both decreasing after 24 hours stimulation. NALP3 exhibited the earliest upregulation, demonstrating a later (after the 6th hour) increase that suggested a possible bi-phasic response.

We found that NOD1 and NOD2 induction upon cognate ligand stimulation or cross-talk stimulation was NALP3 dependent. Silencing of Nlrp3 resulted in significant reduction of *Nod1*/NOD1, *Nod2*/NOD2 and Nlrp3/NALP3 expression in both mRNA and protein levels in unchallenged Sertoli cells (Fig. 1g–i). Upon iE-DAP or MDP ligand challenge for 6 hours after preliminary Nlrp3 silencing, the expression of *Nod1*/NOD1, *Nod2* and Nlrp3/NALP3 was recovered almost to that of non-silenced levels, but never as high as in non-silenced conditions. NOD1 specific challenge had stronger recovery effects compared to NOD2, including more pronounced *Nod2* recovery, suggesting a stronger cross-talk. A co-stimulation of both NOD1 and NOD2 caused induction of *Nod1*, but not of *Nod2*, on mRNA level. *Nod1* mRNA was increased only two-fold under Nlrp3 silenced conditions (Fig. 1g), compared to more than 128-fold under non-silenced conditions (Fig. 1a). We confirmed this re-expression of NOD1 in Nlrp3 silenced conditions at protein level as well (Fig. 1h).

We further investigated if NLR ligands iE-DAP and MDP signal through NFκB pathway in Sertoli cells. Stable Sertoli cell line harbouring NFκB live cell reporter (pNifty2-SEAP), responding only to active NFκB complexes was challenged with iE-DAP, MDP, or a combination of both, for 6, 24 or 48 hours. MDP activated NFκB only after 24 hours of incubation, while iE-DAP had a fast response that lasted for over 48 hours (Fig. 1j). The combined action of both ligands, though cumulative, was relatively short-lived, decreasing to about half of the signal between the 6^th^ and the 24^th^ hour of incubation.

We also challenged TLR4 in adult Sertoli cells for 24 hours using LPS, its specific ligand, and found NALP3 over-expression on mRNA and protein levels (Fig. 1k,l).

### NRL ligands upregulate pro-IL-1β, but only specific priming conditions induce IL1β release in Sertoli cells

**Figure 2:**
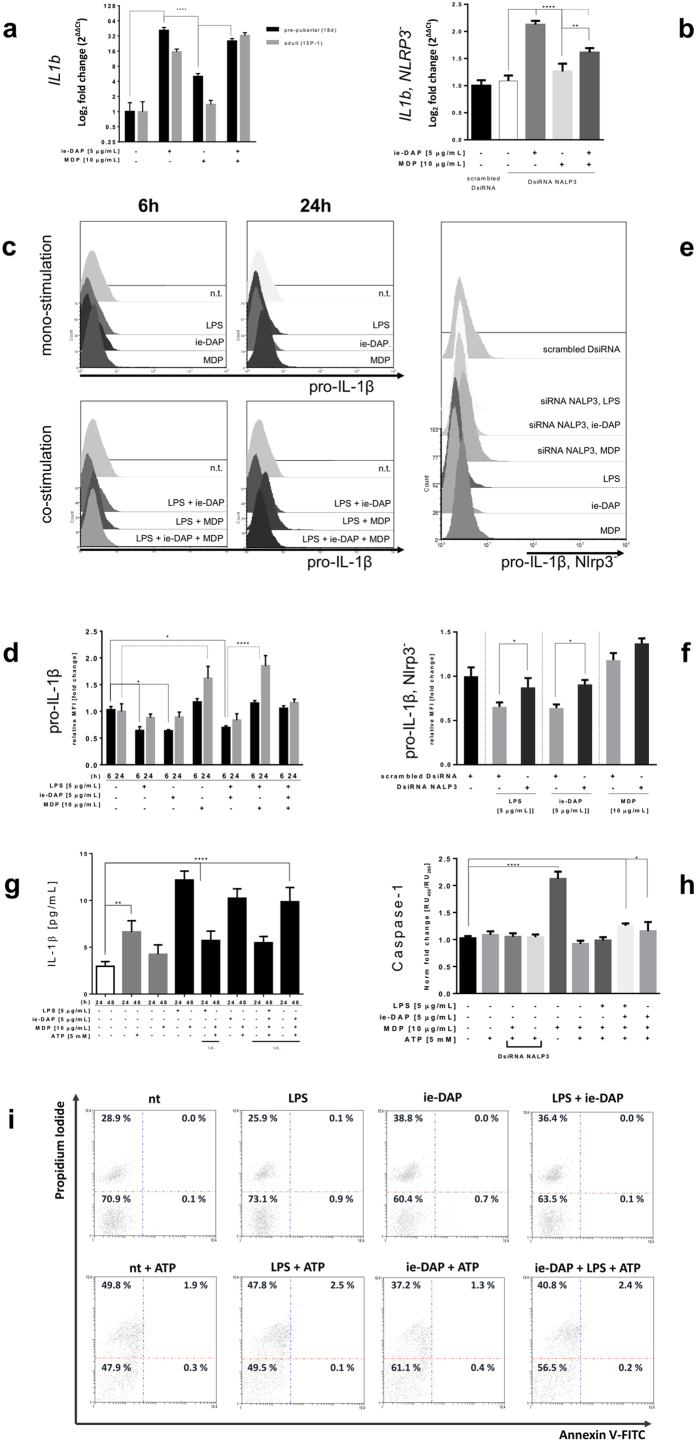
NRL ligands upregulate pro-IL-1β, but only specific priming conditions induce IL1β release in Sertoli cells. (**a**) Quantitative PCR assessment of mRNA transcripts encoding mouse full-length IL-1β proform in primary (black bars) and adult (grey bars) Sertoli cells after challenge (24 h) with iE-DAP and MDP. (**b**) DsiRNA Nlrp3 silencing (72 h) followed by similar challenge (24 h). Data are expressed as log_2_ fold change of non-treated controls compared to challenged ones. Error bars indicate SD. Data are representative of three independent experiments with three technical replicates either using primary Sertoli cells of two isolations (n = 80) or adult Sertoli cell line 15 P-1. (**c**) Intracellular cytokine flow cytometry assessment of pro-IL-1β abundance in 15 P-1 cells subject to challenge with LPS, iE-DAP and MDP for 6 h or 24 h; (**e**) pro-IL-1β flow cytometry assessment after DsiRNA Nlrp3 silencing (72 h) followed by similar ligand challenge (6 h). Gated and preliminary compensated events were acquired and represented as 3D overlaid histogram of event counts vs. channel log fluorescence. (**d**) Intracellular flow cytometry analysis of pro-IL-1β expression in 15 P-1 adult Sertoli cell line after treatment with LPS, iE-DAP, MDP. (**e,f**) Expression is evaluated by the MFI indexes fold induction from the histogram overlay analysis (**c,d**); (**g**) Bioassay quantification of secreted IL-1β (cell media) and (**h**) specific caspase-1 activation assay after ligand challenge (LPS, iE-DAP, MDP) and optionally ATP supplemented (1 h or 24 h). IL-1β assessment in cell lysates (i.c.); (**i**) 2D density plot of flow cytometry assessed Annexin V-FITC (x-axis) and Propidium Iodide (y-axis) expression of Sertoli cells challenged with LPS, iE-DAP and LPS with iE-DAP for 24 h, and ATP for 1 h, compared to control cells (nt). Data are representative of three independent experiments with three technical replicates; Error bars indicate SD; ****P < 0.0001; **P < 0.005; *P < 0.01.

We examined to what extent the inflammasome signalling, as epitomized in inflammatory cells, is enabled in epithelial phagocyting cells. Stimulation of pre-pubertal Sertoli cells with either iE-DAP or MDP resulted in a significant upregulation (over 40-fold) of *Il1b* mRNA. iE-DAP stimulation of adult Sertoli cells gave rise to a 15-fold increase in *Il1b* mRNA, whilst, MDP challenge resulted in only slight induction. The combined iE-DAP and MDP stimulation led to similar significant *Il1b* mRNA upregulation in both pre-pubertal and adult Sertoli cells (Fig. 2a). The induction of *Il1b* mRNA upon NOD1 or NOD2 stimulation was found to be NALP3 dependent. This effect was manifested only upon iE-DAP or combined iE-DAP with MDP challenge, but not under *Nlrp3* knock-down *per se* (Fig. 2b).

Expression of IL-1β proform (pro-IL-1β) in Sertoli cells was studied using intracellular flow cytometry, after cytokine secretion block. The pro-IL-1β expression decreased to almost one half after 6 hour cell stimulation with LPS and iE-DAP, alone or in combination. This reduction diminished after a 24-hour challenge. By contrast, MDP stimulation for 6 hours increased pro-IL-1β expression, almost doubling it after 24 hours of challenge. Simultaneous LPS and MDP stimulation resulted in a similar pro-IL-1β upregulation pattern. Surprisingly, when LPS-mediated TLR4 stimulation was combined with stimulation of both NOD1 and NOD2, the upregulation effect of MDP on pro-IL-1β was antagonistically abrogated, most likely “*overriding*” the MDP effect, thus resulting in neither up- nor down-regulation (Fig. 2c,d). *Nlrp3* silencing partially abrogated the pro-IL-1β reduction, achieved after 6 hours of LPS or iE-DAP stimulation. Similarly, pro-IL-1β expression was further increased after MDP challenge (Fig. 2e,f).

IL-1β secretion by Sertoli cells required specific preliminary ‘*priming’* for 24 hours with either LPS or iE-DAP. Two configurations resulted in IL-1β secretion: TLR4, followed by a NOD2/NALP3 activation, mediated only by MDP, or NOD1, followed by combined MDP and ATP activation of NALP3 (Fig. 2g). In contrast, the 48 hour combined challenge of both NOD receptors, with or without TLR4, although inducing a high amount of intracellular pro-IL-1β in the presence of ATP, did not result in its release. Combined challenges within a 24 hour span could not induce IL-1β secretion (Fig. 2g).

Direct stimulation of NALP3 for 24 hours by MDP resulted in activation of caspase-1, as evident from the caspase-1 activity specific bioassay, and its *Nlrp3* silencing abrogation (Fig. 2h). Combined challenge using either LPS, iE-DAP and MDP, or iE-DAP and MDP, followed by ATP supplement also resulted in caspase-1 activation (Fig. 2h). Additionally, iE-DAP challenge, but not LPS resulted in an increased cell death (Fig. 2i). LPS combination with iE-DAP resulted in an increase compatible with iE-DAP alone. Supplying ATP to LPS/iE-DAP challenge additionally increased the ratio of cell death. Interestingly, 24 hour ATP stimulation, a pre-requisite to induce autophagy, was also able to abrogate MDP-induced caspase-1 activation (Fig. 2h).

In addition, other cytokines, including IL-6 and IL-23, were found to be differentially impacted by NLR signalling. TLR4/NOD1 induced, while NOD2/NALP3 restricted regulation of IL-6, as seen in Supplementary data (Supplementary Fig. 6). Conversely, TLR4 restricted, while NOD1/NOD2 induced IL-23 (Supplementary Fig. 7).

### Proteomics reveals TLR4 and NOD1 to differentially activate autophagy and innate immunity pathways

**Figure 3:**
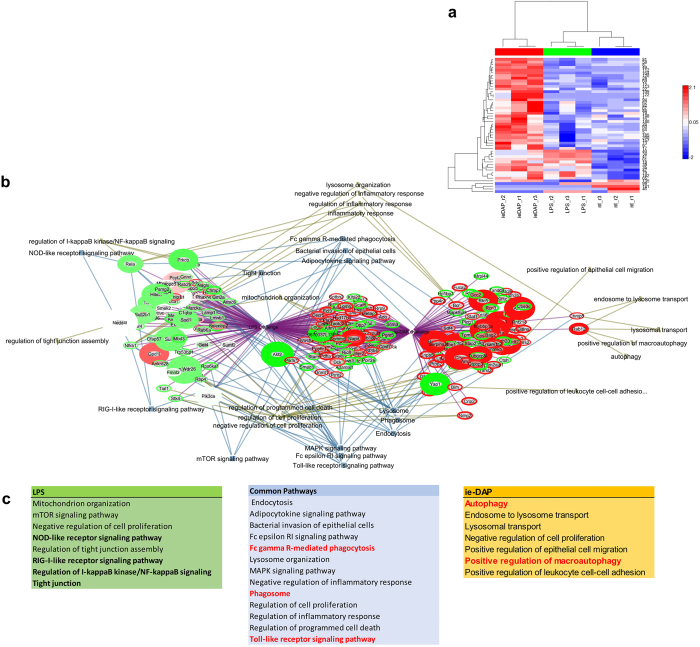
TLR4 and NOD1 specific stimulation differentially activate autophagy and innate immunity pathways. (**a**) Heat map representing log2-transformed, LOESS normalized expression in pairwise comparisons between Sertoli cells challenged with LPS, iE-DAP vs. controls. The top dendrogram represents unsupervised hierarchical clustering using a Complete Linkage Correlation function. The left dendrogram clusters the individual proteins that were selected as significantly different between the treatment conditions in ANOVA statistical analysis using a Median linkage Correlation function. Total proteins detected among treatment conditions lysates were 2295, from which 135 were FDR significantly different in the iE-DAP challenged group and 129 were FDR significantly different (q < 0.001) in the LPS challenged group; (**b**) *EGAN gene enrichment and network linkage analysis* created hypergraph of differentially expressed proteins detected using LTQ Orbitrap MS/MS proteomics of lysates of Sertoli cells challenged with LPS or iE-DAP (24 h), meta-data coincidence calculation annotated signalling pathways and GO Process terms. EGAN enrichment of proteins involved in pathways are shown, probability is depicted by the thickness of gene borders, upregulation is coloured red and downregulation is coloured green; (**c**) Significantly enriched for pathways proteins (EGAN) were grouped in Common Pathways (for both challenges – LPS and iE-DAP) and LPS or iE-DAP specific Pathways lists.

**Figure 4:**
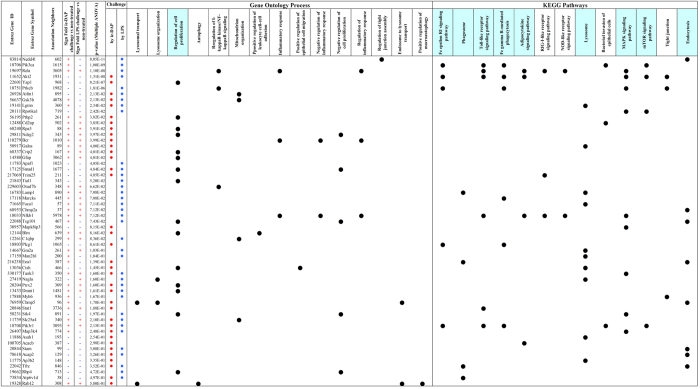
TLR4 and NOD1 differentially altered expression of proteins involved in multiple gene ontology classified processes and signal pathways. Proteomics detected proteins (EntrezGene ID), differentially expressed (LOESS normalized, ANOVA multiple comparison, p < 0.001 filtered) enriched using EGAN and related to GO Process and KEGG Pathways (black circle marked) are presented. Sign of Protein expression Fold change (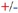) and Challenge (iE-DAP, LPS) that incurred significant expression (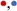) are shown.

We analysed the changes in Sertoli proteome after 24 hours challenge with either LPS or iE-DAP in standard concentrations of 5 μg/mL using LTQ Orbitrap MS/MS proteomics (Fig. 3a, Supplementary Fig. 8). We clustered protein changes according challenge type and we found iE-DAP to predominantly upregulate, while LPS to downregulate proteins. Based on 2295 significantly expressed proteins, from which 135 were FDR significantly different in the iE-DAP challenged group and 129 were FDR significantly different (q < 0.001) in the LPS challenged group, we constructed an EGAN-created gene enrichment and network linkage analysis hypergraph of differentially expressed proteins, meta-data coincidence calculated KEGG signalling pathways and GO Process terms (Fig. 3b,c). Proteins enrichment in pathways was divided to proteins commonly regulated and such specifically regulated by either LPS or iE-DAP. Significantly enriched pathways (EGAN) were grouped in Common Pathways (for both challenges – LPS and iE-DAP) and LPS or iE-DAP specific Pathways lists. Commonly enriched KEGG Pathways to mention were Regulation of programmed cell death, Lysosome organization, Fc gamma R-mediated phagocytosis and Phagosome, while LPS enriched specifically mTOR signalling, NFκB and Tight Junction pathways. iE-DAP on the other hand enriched Autophagy, Lysosomal transport and Positive regulation of macroautophagy (Fig. 3c). Challenging either of the ligands enriched the Toll-like receptor signalling pathway, while LPS specifically enriched the NOD-like and RIG-I-like receptors signalling, supporting experimentally observed cross-talk earlier. Most proteins which expression was significantly altered by LPS/iE-DAP challenge contributed to Cell proliferation, Lysosome and Endocytosis pathway enrichment (Fig. 4).

### Following distinct cellular response reprogramming, NOD1 directs autophagy modulation, while TLR4 directs the assembly of an inflammasome

**Figure 5:**
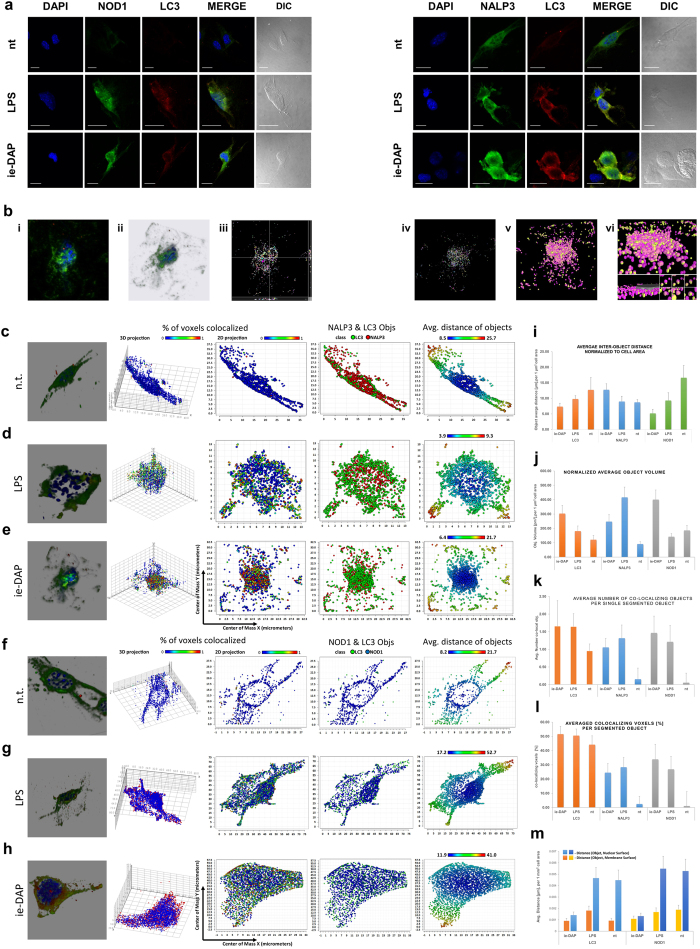
Following distinct cellular response reprogramming, TLR4 directs autophagy modulation, while NOD1 directs the assembly of an inflammasome. (**a**) Confocal immunofluorescence imaging of NOD1 or NALP3 with LC3 in adult Sertoli cells (15 P-1) challenged with LPS or iE-DAP (24 h). Nuclei stained with DAPI. (**b**) *i-vi.* Image segmentation and 3D object analysis of laser-scanned confocal z-stack images of differentially treated 15 P-1 Sertoli cells were subject to noise reduction, deconvolution (b *i-ii*), 3D object segmentation (b *iii*, orthogonal 2D projection; b *iv-v*, 3D volumetric and surface plots), and subsequent object co-localization (b *vi*). Cell and nuclear surface and volume were determined and the cell surface was used as the normalization parameter in distance calculations. Images of the co-compartmentalization (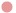) concept of NALP3 (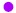) and LC3 (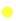) are presented (**b** *vi*); (**c–h**) Panels of analysis data for intact (**c,f**), LPS (**d,g**) and iE-DAP (**e,h**) challenged 15 P-1 Sertoli cells (24 h). Data are represented as a group of a 3D synthetic deconvoluted image, 3D scatter co-localization chart, 2D projection scatter charts of voxel co-localization density, NOD1/NALP3 to LC3 co-localization, object distance; (**i–m**) Normalized summary statistics for average object distance (**i**), volume (**j**), voxel co-localization percentage per segmented object (**k**), average number of co-localizing objects to segmented object for NALP3, NOD1 and LC3 are presented as bar charts (**l**), object average distance to nuclear surface and cell surface estimated for NOD1 and LC3 (**m**). Data are mean ± SD.

We further explored LPS or iE-DAP stimulation impact on the spatio-temporal distribution of NOD1 receptor and the inflammasome scaffold NALP3 in relation to the mature autophagosome marker LC3. Based on the significant level of co-localization of those molecules with LC3 (Fig. 5a) we conducted a 3D object volumetric co-compartmentalization image analysis (Fig. 5b: i-vi, Supplementary Figs 9–11) as described elsewhere^19^. This image analysis approach allows the spatial dynamics of the individual molecular complexes and their co-compartmentalization to be examined. 3D high-resolution segmentation imaging revealed that 24 hours after LPS and iE-DAP challenge, NALP3 (Fig. 5c–e) and NOD1 displayed distinct redistribution and co-localization patterns in conjunction with LC3 (Fig. 5f–h). Based on the object distribution by distance, volume and intensity (Supplementary Fig. 11) and the average object distance, volume and percentage of voxel co-localization patterns (Fig. 5i–m), two types of interaction could be inferred: (i) TLR4 signalling induces NOD1 and NALP3 redistribution, where NALP3 complexes accumulate and aggregate, and their co-localization with LC3 modulates the autophagy process in Sertoli cells without direct NOD1 receptor activation (no NOD1 aggregation); (ii) iE-DAP binding to NOD1 might cause them to dimerize (micro-clustering) and relocate closer to nuclei (Fig. 5m), co-localizing to clustered LC3 complexes, potentially also modulating autophagy. The stimulation also resulted in NALP3 particle size increase and scattered spatial distribution. The latter phenomenon probably reflects the initial stage of inflammasome assembly.

### PCA reveals an iE-DAP responsiveness along the autophagy pathway at individual molecular complex resolution, distinct from that induced by LPS

**Figure 6:**
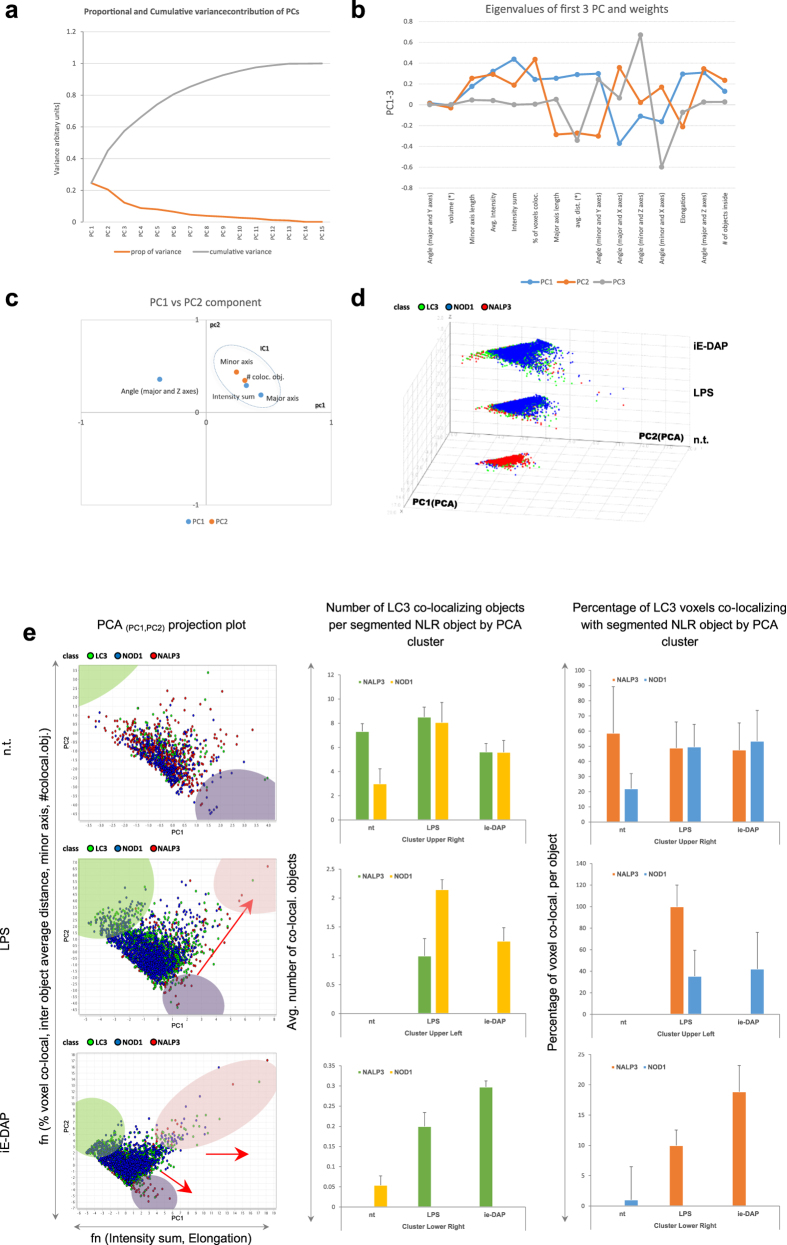
PCA reveals iE-DAP challenge-modulated NLR and LC3 clustering at molecular complex level. (**a**) Variance plot (scree) of the principle components (PC_i_) resulting from PCA of 12 segmentation analysis attributes show 6–7 to account for about 80% of the cumulative variability, with PC1-4 being the most detrimental. (**b**) Eigenvalues of highest variance impeding principle components PC1-PC3 are plotted against the object segmentation attributes, showing those attributes that have highest influence on each principle component. (**c**) Segmentation attributes evaluation by attribute 2D chart plot using principle component eigenvalues as PC space coordinates. (**d**) 3D scatter chart of PC1 (x) vs. PC2 (y) vs. treatment conditions (z), colour coloured by molecule class (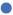 NOD1; 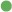 LC3; 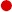 NALP3). (**e**) Selection of individual iE-DAP challenge-produced clusters (provisionally depicted in colours: 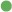
—upper left, 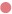
—upper right and 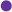
—lower left) from the entire dataset are visualized by treatment modality-dependent 2D scatter projection principle component plots. Voxel co-localization density and object co-localization events intensity per segmented NLR particle and LC3 are presented as individual cluster representative bar charts. Data are expressed as mean ± SD.

To obtain additional insight into the molecular associations of NOD1, NALP3 and LC3, a PCA of the segmented particles derived from the confocal z-stack images was carried out. The PCA procedure derived a set of linearly uncorrelated ‘principal components’ (PC), and we analysed the data for clustering using the leading pair of PC by examining the projections of the stained molecules grouped according the different cell treatment (Fig. 6a–d, Supplementary Figs 12,13). LPS induces relocation of the NALP3 scaffold molecules (projection along PC2) and changes the co-localization of NOD1 and LC3 molecules (projection along PC1) (Fig. 6e, Supplementary Fig. 14). The objects responded to iE-DAP treatment. This responsiveness can be broken down into three modalities that align with the PCs: activation (intensity sum) and redistribution (average object distance) of part of the NOD1 receptors, and LC3 redistribution and co-lateral activation (average object distance, percentage of voxel co-localization), as a marker of an activated autophagy. In parallel, some NALP3 scaffolds underwent aggregation, along with co-localized LC3 molecules. The PCA analysis of 3D segmented data revealed an underlying iE-DAP responsiveness along the autophagy pathway. In addition, we observed that a few NLR molecular complexes per cell were constantly co-localizing with LC3 (Fig. 6e, bar charts).

### TLR4 and NOD1 specific stimulation induce autophagosome assembly and autolysosome fusion

**Figure 7:**
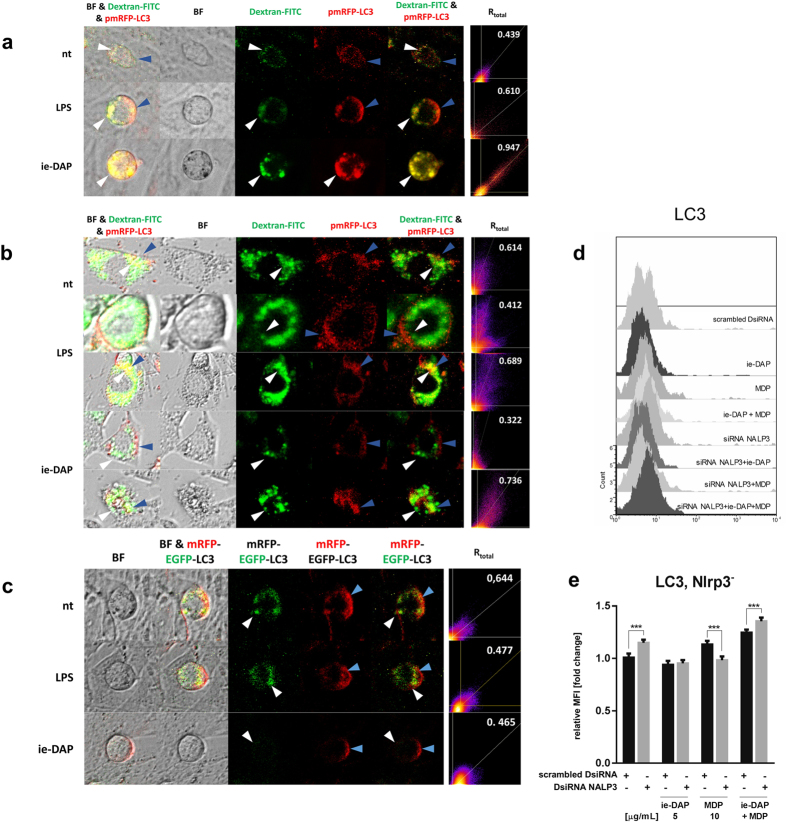
TLR4 and NOD1 specific stimulation induce autophagosome assembly and autolysosome fusion. (**a,b**) Sertoli cells (15 P-1) preloaded with 1 mg/ml FITC-Dextran overnight (37 ^o^C, 5% CO_2_) were transfected with pmRFP-LC3 (24 h), and maintained in either regular DMEM (**a**) or starved for 4 h in serum-free, low glucose DMEM (**b**), and subsequently challenged with LPS or iE-DAP. Chloroquine was added for 3 h and the cells were imaged live on ZOE Fluorescent Cell Imager. Colocalization of lysosomes (Δ) and autophagosomes (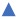) is shown in fluorescent and bright field images (BF). (**c**) Non-starved Sertoli cells (15 P-1) were transfected with tfLC3 encoding pmRFP-EGFP-LC3 (24 h) and challenged with LPS or iE-DAP and subject to live cell imaging. Colocalization of green EGFP incorporated autophagosomes (Δ) and red mRFP autolysosomes (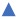) is shown in fluorescent and bright field images (BF). Colocalization ratio (**a–c**) is estimated using ImageJ Colocalization module (Pearson’s correlation coefficient after Costes automatic channel threshold) as R_total_. (**d**) Intracellular cytokine flow cytometry assessment of LC3 abundance in 15 P-1 cells subject to challenge with LPS, iE-DAP and MDP for 24 h; DsiRNA Nlrp3 silencing (72 h); Gated and preliminary compensated events were acquired and represented as 3D overlaid histogram *of* event counts *vs*. channel log fluorescence. (**e**) Expression is evaluated by the MFI indexes fold induction from the histogram overlay analysis.

We investigated the autophagic flux in Sertoli cells following the accumulation of lysosomes and autophagosomes first and then the formation of autolysosomes between them. Autophagosomes and lysosomes accumulation increased after LPS and especially after iE-DAP challenge of FITC Dextran preloaded adult Sertoli cells in non-starved conditions (Fig. 7a). This was marked by co-localization of red fluorescence RFP-tagged mature LC3 (mRFP-LC3) incorporation in forming autophagosomes with Dextran marked lysosomes after LPS and iE-DAP challenge. Under starving conditions (low glucose media) used to trigger autophagy activation, LPS and iE-DAP challenged Sertoli cells formed two populations, one similar to that observed in non-starving conditions, and a low co-localization population, where lysosomes formed perinuclear ring surrounded by partially overlapping peripherally distributed LC3 positive autophagosomes. This difference was especially evident after iE-DAP challenge (Fig. 7b).

We further followed the autophagosome dynamics resulting in autolysosomes formation under TLR4 and NOD1 ligand challenge. Under non-starving conditions Sertoli cells were challenged for 24 hours with either LPS or iE-DAP and transfected with mRFP-GFP-LC3 tandem-tagged fluorescent protein (tfLC3) and subjected to live cell imaging using ZOE (Fig. 7c). The tfLC3 distinguished autophagosome from autolysosome formation based on the differential sensitivity of GFP and mRFP to the lysosomal environment^20^. The mRFP and GFP signals showed different distribution patterns. Although GFP signals colocalized with mRFP puncta in the cytoplasm, a substantial population of the mRFP puncta was found alone, especially away from the perinuclear region. This was most prominent after iE-DAP challenge showing the highest phagosome activation of forming autolysosomes.

We also followed the effect of iE-DAP and MDP challenge on endogenous LC3 expression levels in Sertoli cells. MDP or iE-DAP alone had no significant effect, while their combination increased the LC3 expression (Fig. 7d,e; ATG16L data in Supplementary Fig. 15), but not in NALP3 dependent manner. This data combined suggested an effect of NOD1 and TLR4 singling on the autophagy dynamics, but not on autophagy *de novo* expression.

## Discussion

NOD-like receptors are pivotal for innate host immunity and provide an adequate defense mechanism, linking it to adaptive immunity. While many TLRs are present in mouse Sertoli cells^4^, in rats (*Rattus norvegicus*) only NOD1 mRNA is expressed^3^. However neither NALP3, nor NOD2 have been found in rat Sertoli cells^3^,^21^. The absence of NALP3 and NOD2 is probably related to the observation that NALP3 is found only in cell lineages directly involved in immune responses^22^. However, this view is changing, as NALP1 and NALP3 have been found in different human epithelial cells^23^, including in cells of the testis^24^, making translational impact of this research imminent. In recent studies LPS treatment induced *Nlrp3* mRNA in the testis of C3H/HeN mice^21^. Further, in a mutant mouse strain, LPS induced *in vivo* IL-1β secretion in the testis, thus implying a potential caspase-1 activation pathway^25^.

We have shown that pre-pubertal (primary) and adult (15P-1 cell line) Sertoli cells from BALB/c mice expressed *Nod1*, *Nod2* and *Nlrp3* transcripts and also functional NOD1 and NOD2 receptors and the scaffold protein NALP3 of the inflammasome. Their regulatory significance is supported by other studies reporting that *Nlrp3* and *Nod2* are able to follow a non-canonical 5′-end alternative splicing, exhibiting tissue-specific isoforms^26^.

TLR4 induces NOD1 in bone marrow derived macrophages^27^. We found that upon activation TLR4 did not induce NOD1 and NOD2 in Sertoli cells, but it induced NALP3. Based on the observed NLR differential NFκB dynamics and cross-talk we hypothesize than NOD1 and NOD2 activate different downstream signalling. Our high resolution proteomics data further supported TLR4/NOD1 cross-talk suggesting that either receptor stimulation modulates TLR pathways member expression, while TLR4 specific stimulation directly modulated members of NOD-like receptor and RIG-I-like receptor families. We found that in Sertoli cells NLR stimulation is able to trigger the first phase of the cytokine response, the up-regulation of pro-IL-1β mRNA. Interestingly, with cell maturation, NOD2 lost the ability solely to stimulate IL1b gene expression, thus limiting the chance for prolonged IL-1β over-expression.

More importantly, in Sertoli cells NLR activation, especially of NOD1, was able to restore *Nalp3* mRNA levels, after preliminary siRNA-mediated functional silencing, suggesting the induction of its synthesis *de novo*. The priming and subsequent NALP3-mediated activation of caspase-1 in macrophages was also shown to be functionally limited by *de novo* protein synthesis, being regulated by either MyD88- or TRIF-mediated *Nlrp3* transcript accumulation^28^.

Recently, Martinon and Tschopp (2007) proposed a ‘*two-signal’* model of inflammasome activation. This advocated that NALP3 expression is restricted in dendritic cells (DCs) and macrophages and that the *Nlrp3* gene promotor can only be primed by TLR4 or NOD1 activation. This priming provides the necessary NFκB-mediated transcriptional induction of cytokine precursors, such as pro-IL-1β. In a second, post-transcriptional step, the NALP3 inflammasome becomes activated. Only then, if caspase-1 becomes active, it produces the mature cytokine^29^,^30^,^31^.

We found that TLR4/NOD1 challenge reduced protein levels of pro-IL-1β in contrast to NOD2/NALP3 challenge. Considering the secretion of IL-1β, it is likely that TLR4/NOD1 reduction of the proform in first 6 hours up to 24 hours could be a result of active cleavage rather than reduced expression, especially considering the concomitant mRNA upregulation of the same IL1B gene. The pro-IL-1β protein expression reduction was NALP3 dependent, as silencing of Nlrp3 abrogated it at 6 hours challenge of TLR4/NOD1, but not when NOD2/NALP3 was directly challenged. In contrast, MDP stimulation alone resulted in upregulation of pro-IL-1β expression, most likely due to decreased cytokine cleavage, as secreted form was reduced. Functional silencing revealed that NALP3 differentially modulated NLR cross-talk and NLR-induced *Il-1b* transcriptional activation, significantly promoting NOD1-, but limiting NOD2- signalling. *Nlrp3* silencing limited TLR4/NOD1-mediated pro-IL-1β reduction, but not NOD2- signalling.

In consideration of the observation that pro-IL-1β reduction in TLR4/NLR ligand challenged Sertoli cells, we questioned whether this was a result of cleavage^31^ or represented a regulatory step^32^. To address this, we followed the cleaving activity of caspase-1 and secretory pattern of IL-1β as the latter could reflect an early reduction in pro-IL-1β.

Inflammasome activation is required for that of caspase-1. In macrophages, NALP3 is activated by a combination of MDP and a second substance such as ATP^8^. NOD2 engagement of MDP is also required for ATP priming^33^. Extracellular ATP serves as a damage-associated molecular pattern (DAMP) signal, and could act via complex of purinergic ATP-gated P2 × 7 receptor (P2 × 7R) and ATP-activated Pannexin-1 pore (Panx1). Both P2 × 7R and Panx1 are required for ATP-induced potassium efflux and were found to be expressed in Sertoli cells at the level of the blood-testis barrier^34^,^35^. Observing different time and ligand combinations, we conclude that IL-1β secretion in Sertoli cells requires preliminary ‘*priming’* for 24 hours with either LPS or iE-DAP. This must be followed by NALP3 activation mediated via MDP with or without ATP. We suggest that in scenarios where ATP is required, it is most likely a second signal that triggers the P2 × 7R/Panx1 axis to activate the NALP3/caspase-1. Unlike Sertoli cells, mouse DCs could over-express cytokine genes upon MDP challenge and inflammasome activation with uric acid in a NALP3-independent manner^36^. MDP alone induced a small amount of IL-1β secretion in this cell type (Sertoli), and this was ATP- and priming-independent, but caspase-1 mediated. Surprisingly, ATP alone was also able to produce even higher levels of IL-1β in Sertoli cell media, although there was no caspase-1 activation. We suspect that this could be cell-death related phenomenon, since ATP increased cell death and the detected IL-1β in cell media could be released as a pro-form (pro-IL-1β).

We suspect that Sertoli cells require the NALP3 inflammasome for a physiological source of IL-1β to deliver IL-1α^37^. This is supported by the low levels of secreted IL-1β and the detection of intracellular IL-1β after 6 or 24 hours of NLR challenge that is not further secreted in cell culture media. Hence, neither the pro-IL-1β-limiting TLR4\NOD1 nor the pro-IL-1β-inducing NOD2\NALP3 functions pertain to mature IL-1β secretion. On the other hand, in case of secretable IL-1β upregulation the IL-1β shuttling function would result in an increased IL-1α paracrine secretion, that would “open” the TJ of the BTB as suggested for IL-1α function by Lie *et al.*^38^. When combined with caspase-1 induced cell death, the NLR\NALP3 axis could serve as pathological pathway for male infertility. Cell blebbing and shrinking were also found along the way in an “*all-or-none*” fashion similar to the one described for caspase-1^39^. Extracellular ATP serving as DAMP also potentiated cell death increasing the signalling potential of cell damage. Thus, the release of IL-1 either as pro-IL-1β or mature IL-1β could serve as a pro-inflammatory signal. The released of pro-IL-1b following tissue injury and cell death could be cleaved extracellularly by proteases derived from infiltrating neutrophils resulting in an IL-1b-dependent inflammatory response independent of caspase-1 like the one observed in sterile tissue injury and acute arthritis^40^ . Pro-IL-1b can also be processed into biologically active molecules by some bacterial proteases^41^, suggesting that extracellular processing may also occur at inflammatory lesions caused by infection.

It is likely that autophagy plays an important role in limiting pro-IL-1β processing and secretion, since mature cytokine secretion and caspase-1-specific activation were both abrogated by prolonged ATP treatment (24 hours and more). In BMDM and monocytes, autophagy was found to control the production of IL-1β, either by targeting pro-IL-1β for lysosomal degradation or by regulating the activation of the NALP3 inflammasome^16^. Autophagy inhibition activates the inflammasome and IL-1β secretion in LPS-treated BMDC and iBMM, promoting cytokine processing and secretion. This activity would otherwise be sequestered in LC3-autophagosomes, and thus effectively exclude caspase-1^32^.

Since this cytokine secretion pathway was found to be negatively regulated by ATG16L and ATG7^42^ and *Nlrp3* silencing impaired iE-DAP-mediated pro-IL-1β ‘*clearance’* we investigated if there was an interaction between NLRs and the autophagy pathway. Few data exist regarding how autophagy and NALP3 interact. High-resolution 3D object segmentation imaging has revealed differential TLR4 and NOD1 signalling modalities on NALP3 aggregation and LC3 binding. Since NOD1 dimerization occurred only after binging to its cognate ligand iE-DAP, but not after TLR4-mediated NOD1 relocation and LC3 binding, we suggest that TLR4 initiation might be a first signal, which could lead to the relocation of the endogenous NOD1 receptor in an “armed state” closer to LC3-autophagosome vesicles. PCA analysis of the segmented particles further revealed an underlying iE-DAP responsiveness along the autophagy pathway. Our data demonstrated that iE-DAP binding to NOD1 changed LC3 particle intensity (3D imaging), upregulated autophagy pathway proteins, promoting also endocytosis and lysosomal pathways (proteomics) and increased autophagy flux and autolysosome formation out of autophagosomes (tfLC3 flux study). Our observations are in agreement with recent findings that NOD triggering activates autophagy^43^, which confines intracellular bacteria within autophagosomes. This serves to restrict an infection^44^. Similarly, in Sertoli cells NOD1 promoted autophagy via mature autophagosome fusion to lysosomes and formation of autolysosomes, but not by direct LC3 expression alteration. Travassos *et al.* (2010) found that NOD1 and NOD2 probably serve as molecular scaffolds, physically delivering bacterial pathogens to the autophagy machinery in BMDM by interacting with ATG16L1^18^.

Our data suggest a revised view that Sertoli and possibly other epithelial cells upon pathological “*pro-inflammatory*” conditions are only immunotolerant, i.e. they produce only IL-1α, not IL-1β. Several new findings also support this hypothesis. The receptor tyrosine kinase subfamily Tyro3 (TAM) is responsible for inflammatory response inhibition and the promotion of phagocytosis by DCs and BMDM^45^. TAM was found to restrict the up-regulation and secretion of the inflammatory cytokines IL-1β, IL-6, TNF and type I IFNs in response to challenge of TLR3 in Sertoli cells, as proved by a TAM triple negative mutant mice model^25^. TLR3 was found to dramatically augment NOD1/NOD2- signalling^46^, thus further implicating NOD2 signalling in IL-1β maturation. We also demonstrated pilot data on IL-6 and IL-23 cytokines expression in Sertoli cells upon “*pro-inflammatory*” stimuli, suggesting for further blood-testis barrier impairment and pathogenic IL-23–IL-17 axis activation.

Overall, our data provide a translational mechanistic support for the growing amount of findings that males with infertility have increased IL-18 levels in their seminal plasma, regardless of their urogenital inflammatory status^47^,^48^, as the activation of caspase-1 has been proved sufficient to induce cleaving of both IL-1β and IL-18^49^.

In summary, our study has highlighted the importance of revising the current paradigm that inflammasomes are only present in and required by the professional immune cells as primary drivers of the inflammatory immune response. Here, we demonstrate that Sertoli cells, which are non-immune and immunotolerant, are able to utilize the NALP3 inflammasome and caspase-1 to produce pro-inflammatory IL-1β in the physiological context. Sertoli cells are epithelial non-professional antigen-presenting cells capable of phagocytosis. This distinction could implicate the Sertoli cell in autoimmune and inflammatory scenarios that have yet to be discovered.

## Materials and Methods

### Primary Sertoli cell isolation and culture

Mice (*Mus musculus,* n = 80), of BALB/c background were purchased from the Bulgarian National Animal Facility. The mice were maintained on a normal diet under 12-hour light and dark cycles and specific pathogen-free conditions in the Animal Research Facility at the Institute of Biology and Immunology of Reproduction. Animal maintenance and procedures, and all experimental protocols were in accordance with the institutional Animal Care and Use Regulations, in compliance with the Bulgarian husbandry regulations (Animal Protection Law of 31.01.2012, Decree № 20 of 01.11.2012: “Protection and welfare of experimental animals and requirements to objects of use and cultivation and/or delivery”) and EU Regulations of Animal Use and Care. All experimental protocols were approved by the Animal Care and Use Committee of the Institute of Biology and Immunology of Reproduction.

Sertoli cells were isolated from seminiferous tubules of 18-day-old pre-pubertal males according to a protocol described elsewhere^50^. The isolated cells were cultured in bovine serum albumin (BSA)-free, buffered DMEM:F12 media, supplemented with 1/100 dilution (v/v) of insulin–transferrin–selenium (Sigma), 2.5 ng/mL epidermal growth factor (Sigma), 5 μg/mL bacitracin and 20 μg/mL gentamicin (Genaxxon) and kept in a humidified atmosphere of 5% CO_2_/air, at 32 °C.

The Sertoli cell line 15P-1 was obtained from the ATCC (code: CRL-2618). The cell line was derived from six-month-old adult male mice^51^. The 15P-1 cells were kept under culture conditions as described above and maintained in DMEM tissue culture fluid (Sigma).

### NALP3 DsiRNA transfection

Transfection experiments were carried out using validated Dicer-substrate RNA (DsiRNA), targeting the exon 4 coding sequence of *Nlrp3* (IDT Inc.), utilizing a chemically synthesized RNA duplex technology for enhanced (~100 folds) gene-specific silencing^52^. The transfection protocol (Qiagen) employed HiPerfect reagent and 5 nmol DsiRNA with following sequence: 5′-AAC CUG CUU CUC ACA UGU CGU CUG UAC-3′. Silencing specificity was ensured using 5 nmol DS NC1 universal scrambled transfection control (IDT) and knock-down transfection efficiency was validated using RT-qPCR NALP3 amplification, NALP3 flow cytometry evaluation and death siRNA (Qiagen) parallel transfection experiments.

### Reagents

Muramyl dipeptide (MDP) and γ-D-glutamyl-meso-diaminopimelic acid (iE-DAP) were purchased from InvivoGen. Lipopolysaccharide (LPS) from *Escherichia coli*, serotype EH100 (Ra) (TLRgrade), was purchased from Enzo Life Sciences. Adenosine 5′-triphosphate (ATP; disodium salt) was purchased from Genaxxon. In subsequent experiments LPS and iE-DAP were used at a concentration of 5 μg/ml, MDP was used at a concentration of 20 μg/ml and ATP was used at a concentration of 5 mM.

### Real-time reverse transcription quantitative PCR analysis

Total RNA was isolated from primary or 15 P-1 Sertoli cells previously incubated with various combinations of iE-DAP, MDP and LPS for 24 hours, using RNeasy mini kit (Qiagen). Genetic material in the form of cDNA was synthesized from 1 μg of total RNA using Sensiscript Reverse Transcriptase (Qiagen) kit. Quantitative amplification was performed on a real-time qPCR MX3500P cycler (Stratagene) using EvaGreen Master Mix (Geneaxxon) with the indicated primers (Supplementary Table 1). Expression levels of NOD1, NOD2, NLRP3 and IL-1β were normalized with reference to expression levels of β-actin transcripts as a housekeeping gene, and are represented as a relative fold change showing experimental vs. control gene expression ratio, following the previously described ΔΔCt method^53^.

### Flow cytometry

15 P-1 Sertoli cells were seeded into 6-well plates 24 hours prior treatment and were exposed to combinations of the ligands iE-DAP and MDP at intervals of 1, 2, 4, 6 and 24 hours. Similarly, LPS challenge was conducted for 24 hours. Protein expression of the relevant receptors was assessed using polyclonal anti-mouse antibodies raised in a goat against LC3b (N-20) or NOD2 (P18), or raised in a rabbit against NOD1 (H-176) or NALP3 (H-66). Isotype control antibodies and secondary relevant FITC-conjugated mouse anti-goat IgG and PE-conjugated mouse anti-rabbit IgG were used as a detection system (Santa Cruz Biotechnology).

Intracellular detection of cytokines^54^ was carried out by subjecting cells to 6 hours of specific ligand challenge. The blocking inhibitors Monensin and Brefeldin A (eBiosciences) were used to inhibit endosomal trafficking. They were applied for 3 hours, starting at the third hour following ligand stimulation. Direct conjugated anti-mouse antibodies specific for IL-1β were used to detect the intracellular abundance of the IL-1β proform (FITC conjugated, clone NJTEN3, 0.5 μg/10^6^ cells), all purchased from eBioscience (Supplementary Table 2).

In some experiments, DsiRNA NALP3 transfection was preliminarily conducted for 72 hours. Control and treated Sertoli cells were detached using Accutase™ (eBiosciences) and immersed in cold 1% BSA/PBS/0.1% NaN_3_. The cells were treated using eBiosciences IC fixation buffer, fixation/permeabilization solution for intracytoplasmic staining and flow cytometry staining buffer block. Thereafter, the specific primary or the appropriate isotype control antibodies, at the specified above concentrations, were applied for 30 min at 4 °C, followed by flow cytometry staining buffer wash. The secondary antibody was added at a concentration of 0.25 μg/10^6^ cells for 30 min at 4 °C (in the dark). After washing, cells were gated using forward vs. side scatter, to exclude dead cells and debris, and collected and analysed with a BD FACSCalibur flow cytometer (Becton Dickenson). Fluorescence of 10^4^ cells per sample was acquired in logarithmic mode for visual inspection of the distributions and for quantifying the expression of the relevant molecules by calculating the median fluorescence intensity (MFI) of samples^55^.

Cell death and apoptosis were assessed using Annexin V-FITC and Propidium iodide (PI) using eBioscience standard kit and manufacturer’s protocol.

### Stable Cell Line generation and NFκB SEAP Reporter Assay

The pNifty2-SEAP plasmid (Invivogen) was transfected using Attractene reagent (Qiagen) for 48 hours in 1.10^5^ 15 P-1 Sertoli cells. Following 72 hours, the stimulated Sertoli cells were selected with Zeocin (250 μg/mL, Invivogen) for 5 days and subsequently cultured in the presence of 100 μg/mL Zeocin thereafter. Upon challenge with iE-DAP, MDP, or both, in separate experiments, nuclear factor kappa B (NFκB) activation was monitored live via the secreted alkaline phosphatase (SEAP) release in collected cultivation media using a colorimetric detection (Invivogen), at 405 nm (BMC FLOWStar Optima reader).

### Immunofluorescence staining

Sertoli cells were seeded on LabTek (Sigma) slides and cultured overnight. The cells were then treated with LPS and iE-DAP for 24 hours. The cells were then fixed with 4% (w/v) paraformaldehyde at 4^o^ C, for 20 min, and permeabilized with 0.2% (v/v) Triton-X 100 in PBS for 15 min. The cells were then blocked with 2% (w/v) BSA-PBS for 1 hour. Immunodetection was performed sequentially for each antigen and incubated with the same antibodies used for flow cytometry at a dilution of 1/50 in PBS overnight, at 4 °C, and after three PBS washes they were incubated for 1 hour in the dark, using donkey anti-rabbit IgG antibody conjugated with Alexa Fluor® 488 or donkey anti-goat Alexa Fluor® 555 (Invitrogen) at a dilution of 0.25 μg/100 μl. To detect the cell nuclei, DAPI (Sigma) was used at a 1:250 dilution in PBS. Microscopy slides were prepared with Vectashield mounting medium (Vector Labs) and covered with coverslips sealed with *Marabu Fixogum* rubber cement.

### Confocal microscopy

Microscopy slides prepared for immunofluorescence were subjected to an established image acquisition protocol^56^ using a Nikon A1R laser-scanning confocal microscope equipped with an apochromatic, violet corrected, 60×, 1.4 numerical aperture oil immersion objective (NIS Elements software, Nikon). Three-dimensional (3D) single-cell images were acquired in four channels for differential interference contrast (DIC) and each colour channel (red, green and blue) using one-way sequential line scans. The scanner zoom was centred on the optical axis and set to a lateral magnification of 40 nm/pixel, and a Z-depth with increments of 140 nm.

### D image segmentation and Principle Component Analysis (PCA)

The acquired 3D image stacks (ND2) were converted to OME-TIFF format, aligned using ImageJ plugin StackReg in translational mode. Images were de-convolved by ImageJ plugin DeconvolutionLab, using the algorithm “Richardson-Lucy with TV regularization” (an example is shown on Fig. 5b(i)). Channel-based PSF function images were generated for each acquired image using PSF Generator plugin (http://bigwww.epfl.ch/algorithms/psfgenerator/), “Richards and Wolf algorithm. DIC and fluorescent images were used to create mask binary channels by tracing cell outlines. Automated image processing and analysis pipeline (see the pipeline scheme in Supplementary Fig. 10) was setup in BioImage XD. For each segmented object (Fig. 5b(iii-iv)) a 3D mesh was generated (Fig. 5b(v)) that was used to assess the object co-compartmentalization (further referred to as object co-localization) with other objects (Fig. 5b(vi)) using two parameters: percentage of voxels (3D pixels) co-localizing with the 3D mesh of other objects, and the number of separate co-localizing objects that “overlap” in 3D space with this object, ex. green channel objects overlapping with red channel ones. The mask binary channels corresponding to DAPI nuclear staining and the cell surface were used for polyedric 3D nuclear/cell membrane surface mesh generation used to estimate the distance between the receptor objects and the nuclear/cell surface (Fig. 5vi). Object attributes (attr_1–16_) estimated: percentage of voxels co-localized, number of separate objects co-localizing with a specific object, object average distance, volume, average intensity, intensity sum (an integrated object intensity), elongation, minor axis, major axis, angles between either object major or minor axis and each of the Cartesian axis (x, y, z) (six angles in total), object centre of mass distances to nuclear and membrane surface respectively.

The object segmentation yield per colour channel was more than 8000 separate entities, each described by 16 attributes, thus generating 18-dimensional space (attr_1–16_, treatment [n.t., LPS, iE-DAP], class [NALP3, NOD1, LC3]). We applied PCA to reduce the data dimensionality and inter-correlation, and find those parameters with high impact on the data variability. PCA created new synthetic variables, allowing for data examination and cluster identification. Using data mining suite RapidMiner (Cambridge, MA), subsequent sub-selection of three individual iE-DAP challenge-produced clusters was performed on the entire dataset and separated by treatment modality using Mondrian freeware software.

### Autophagic flux assessment

Sertoli cells (15 P-1) were supplemented with 1 mg/ml FITC-Dextran 4 kDa (Sigma) overnight (37^o^ C, 5% CO_2_). Cells are transfected for 24 h using Attractene and 500 μg pmRFP-LC3 (AddGene, US^20^). Some of the cells were starved for 4 h in serum-free, low glucose DMEM (Sigma). The cells were challenged with LPS or iE-DAP and imaged live on ZOE Fluorescent Cell Imager (Bio-Rad, USA). Other cells were transfected for 24 h using Attractene and 500 μg pmRFP-EGFP-LC3 (ptfLC3) (AddGene, US^20^) and challenged with LPS or iE-DAP. Colocalization ratio was estimated using ImageJ Colocalization module (Pearson’s correlation coefficient) as R_total_ after automatically setting channel thresholds (using Costes method) and estimating the Manders coefficients.

### ELISA

Sertoli cells were plated on a 96-well plate (1–2.10^4^/well) and stimulated with LPS, iE-DAP or MDP for either 24 hours or 48 hours, and in some cases ATP was added to the cultures. Culture fluid was collected for assessment of secreted IL-1β. Cell lysate was prepared for assessment of intracellular expression. The murine-specific bioassay was applied according to the manufacturer’s instructions (Mouse IL-1 beta ELISA Ready-SET-Go! ^®^, eBioscience). The concentrations were estimated by sigmoidal interpolation of a standard dilution assay (GraphPad Prism, v.6) using the optic density readings (λ = 405 nm).

### Caspase-1 Assay

Sertoli cells were seeded in a 6-well plate (2 × 10^5^/well) and stimulated with LPS, iE-DAP, MDP and ATP for 24 hours. Occasionally, DsiRNA NALP3 transfection was preliminarily conducted for 72 hours. Activated caspase-1-mediated specific substrate cleavage was detected using a colorimetric assay, following the manufacturer’s protocol (BioVision). The caspase-1 activity was read on 405 nm (BMC FLOWStar Optima) and estimated as relative activity change of ligand-challenged cells vs. untreated ones. All results were normalized to protein content using parallel readings at 280 nm.

### Quantitative Proteomics

Lysates were obtained from Sertoli cells challenged with either LPS or iE-DAP in standard concentrations of 5 μg/mL for 24 hours. Protein samples were prepared and analysed in triplicate as described previously^57^,^58^. Briefly, the analysis of protein digests by electrospray ionization MS was performed on a hybrid Linear Trap Quadrupole (LTQ)/Orbitrap Velos instrument (Thermo Fisher) interfaced to a split-less nano-scale HPLC (Ultimate 3000, Dionex). The LTQ/Orbitrap Velos was operated in the Top20 data-dependent mode with 2 high-resolution scans (resolution of 30,000 at 400 m/z) followed by 20 MS/MS scans for the 20 most abundant peptide ions having a charge state > 1. All LC-MS/MS data were processed by MaxQuant using the latest International Protein Index (IPI) fasta file for protein identification. Protein label-free intensities as reported by MaxQuant were normalized using the sum of all intensities as a normalizing factor and then used to identify differentially-expressed proteins. The InfernoRDN (http://omics.pnl.gov/software/infernordn) was used for this analysis of log_2_-transformed intensity data, imputed 0 values with 1, ANOVA filtered and LOESS normalized, adjusted p-values estimation using the false discovery rate algorithm^59^,^60^.

**EGAN**: **Exploratory Gene Association Networks** (http://akt.ucsf.edu/EGAN/) was further used on ANOVA filtered proteins for hypergraph visualization of proteins and meta-data enrichment analysis (Gene Ontology annotation, KEGG, PANTHER signalling pathways).

### Statistical Analysis

Data were generated from three independent experiments, each performed in triplicate. One-way or two-way ANOVA tests with respective multiple comparison post-tests (Holm-Sidak’s or Tukey correction and Adjusted P value and Family-wise significance and confidence level of 0.05) were used (GraphPad Prism 6 software). P < 0.01 was considered significant.

## Additional Information

**How to cite this article**: Hayrabedyan, S. *et al.* Sertoli cells have a functional NALP3 inflammasome that can modulate autophagy and cytokine production. *Sci. Rep.* **6**, 18896; doi: 10.1038/srep18896 (2016).

## Supplementary Material

Supplementary Information
