# Supplementary material for: Sertoli cells have a functional NALP3 inflammasome that can modulate autophagy and cytokine production
Source: Sci Rep. 2016 Jan 8;6:18896. doi: 10.1038/srep18896 (PMC4705529; doi:10.1038/srep18896)
Supplement: Supplementary Information [file srep18896-s1.pdf]

SUPPLEMENTARY INFORMATION

Sertoli cells have a functional NALP3 inflammasome that can modulate autophagy and cytokine production

Soren Hayrabedyan[1], Krassimira Todorova[1], Asma Jabeen[2], Gergana Metodieva[2], Stavri Toshkov[3], Metodi V. Metodiev[2], Milcho Mincheff[3], Nelson Fernández[2]

[1] Institute of Biology and Immunology of Reproduction, Bulgarian Academy of Sciences, Sofia, Bulgaria;

[2] School of Biological Sciences, Wivenhoe Park, University of Essex, Colchester, UK;

[3] Cellular and Gene Therapy Ward, National Specialized Hematology Hospital, Sofia, Bulgaria

Supplementary info:

1. Supplementary Table 1. Primer pairs used for RT-qPCR (Table)
2. Supplementary Table 2. Antibodies used in Immunofluorescence imaging and Flow cytometry experiments
3. Supplemental Figure 1 - Primary pre-pubertal Sertoli cells isolated from 18 day old male mice
4. Supplemental Figure 2 - Confocal fluorescence images of prepubertal primary Sertoli cells isolated from 18d BALB/c male mice, under intact or challenged with either ie-DAP or MDP conditions, stained for NOD1, NALP3, ATG16L and LC3
5. Supplemental Figure 3 - Confocal fluorescence images of Sertoli cell line 15P-1 under intact or challenged with either LPS or ie-DAP conditions, stained for NOD1, NALP3 and co-stained for ATG16L.
6. Supplemental Figure 4 - Confocal fluorescence images of Sertoli cell line 15P-1 under intact or challenged with MDP conditions, stained for NOD2. Detail section presents zoomed in portions of the cytoplasm with characteristic NOD2 positive stained granules
7. Supplemental Figure 5 - RT-qPCR data showing that FSH influences NOD1, NOD2, NALP3 mRNA expression in primary Sertoli cells (15d) grown in hormone supplemented media in ie-DAP and MDP challenge conditions
8. Supplemental Figure 6 - Intracellular flow cytometry assessment of protein expression of IL-6: Reciprocal regulation of IL-6 by TLR4/NOD1 and NOD2/NALP3 signalling
9. Supplemental Figure 7 - Intracellular flow cytometry assessment of protein expression of IL-23: TLR4 have suppressive, while NOD1 and especially NOD2 cross-talk most probably have inductive effect on the production of IL-23 by Sertoli cells
10. Supplemental Figure 8 - Proteomics Data Pre-processing
11. Supplemental Figure 9 - Assessment of secondary antibodies non-specific co-localization
12. Supplemental Figure 10 - Full workflow from Image acquisition to segmented objects statistical analysis
13. Supplemental Figure 11 - Segmented objects distribution by Obj. Avg. Distance or Obj. Avg. Intensity (x) plotted against Obj. Avg. Volume and grouped by treatment modality, presented using 3D scatter plot.
14. Supplemental Figure 12 - RapidMiner data flow of the analyses performed (PCA and ICA) on the CSV files exported from BioImage XD containing segmented objects image data
15. Supplemental Figure 13 - Variance plot (scree) of the principle components showing those with highest impact on the total variance. Several principle component 2D charts plotting each original attribute against selected principle components, with object attributes eigenvalues representing abscise and ordinate (x,y) values respectively.
16. Supplemental Figure 14 - Use of Mondrian software for analysis of three subpopulations defined using PCA
17. Supplemental Figure 15 - Intracellular cytokine flow cytometry assessment of ATG16L abundance in 15P-1 cells subject to challenge with LPS, iE-DAP and MDP for 24h; DsiRNA Nlrp3 silencing

Supplementary Table 1. Primer pairs used for RT-qPCR

Gene ID	Forward Primer	Reverse Primer
Nod1	5’-CTCACGGTTATCAGACTCAGC-3’	5’-TTCCTCACATAGCACCTTCAC-3’
Nod2	5’- TGGCCAGGATTCTGAAGGGTTC-3’	5’-ATCCACAGCAACAGGCGTTCAG-3’
nlrp3	5’-ACTGACCATCCCGCATAAGGAG-3’	5’- TGAGAGGCTGCCACAAACCTTC-3’
il1b	5’-CAAGGCCACAGGTATTTTGT-3’	5’-GAAATGCCACCTTTTGACAG-3’
actb	5’-CTGGGAGTGGGGAGGC-3’	5’-TCAACTGGTCTCAGTG-3’

Supplementary Table 2. Antibodies used

Antibody	Clone	Vendor	Used concentrations [µL]	Channels used
Anti-Mouse IL-1 beta Pro-form FITC	NJTEN3	eBioscience Inc.	0.5 µg/10^6 cells	FL1 / FITC
Anti-Mouse IL-6 PE	MP5-20F3	eBioscience Inc.	0.2 µg/10^6 cells	FL2 / PE
Anti-Mouse IL-10 APC	JES5-16E3	eBioscience Inc.	0.125 µg/10^6 cells	FL4 / APC
Anti-Mouse IL-23 p19 eFluor® 660	fc23cpg	eBioscience Inc.	0.06 µg/10^6 cells	FL4 / APC
Anti-Mouse ATG16L	C-20	SCBT	0.5 µg/10^6 cells	FL1 / FITC
Anti-Mouse LC3b	N-20	SCBT	0.5 µg/10^6 cells	FL1 / FITC
Anti-Mouse NOD1	H-176	SCBT	0.5 µg/10^6 cells	FL2 / PE
Anti-Mouse NOD2	P-18	SCBT	0.5 µg/10^6 cells	FL1 / FITC
Anti-Mouse NALP3	H-66	SCBT	0.5 µg/10^6 cells	FL2 / PE
isotype mouse IgG	sc-3877	SCBT	0.5 µg/10^6 cells	
isotype rabbit IgG	sc-3888	SCBT	0.5 µg/10^6 cells	
isotype goat IgG	sc-3887	SCBT	0.5 µg/10^6 cells	
mouse anti-goat IgG-FITC	sc-53800	SCBT	0.25 µg/10^6 cells	
mouse anti-rabbit IgG-PE	sc-3753	SCBT	0.25 µg/10^6 cells	
donkey anti-rabbit IgG (H+L) Alexa Fluor® 488	A-21206	Life Technologies / ThermoFisher Scientific	0.25 µg/100 µl	green channel (confocal)
donkey anti-goat IgG (H+L) Alexa Fluor® 555	A-21432	Life Technologies / ThermoFisher Scientific	0.25 µg/100 µl	red channel (confocal)

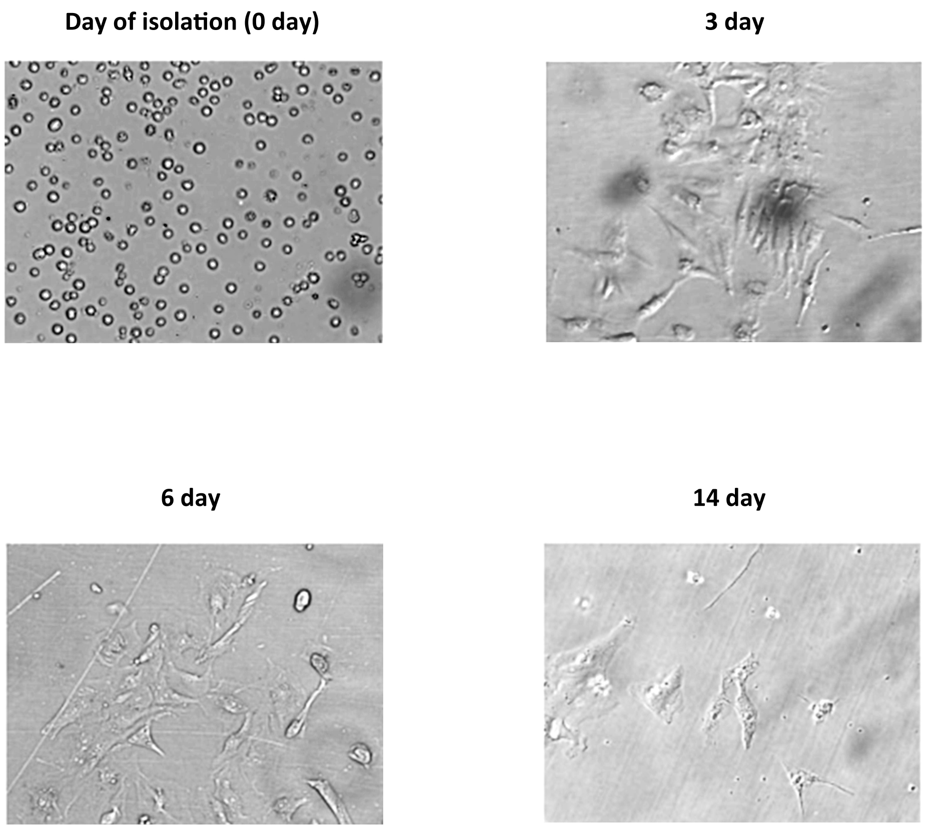

Supplemental Figure 1. Primary pre-pubertal Sertoli cells isolated from 18 day old male mice (n=80).
Samples are shown on the day of isolation and after 3, 6 and 14 days.

The experiments were done at 3-6 day, as at day 14 the cells acquired senescence phenotype, with giant cell formation and vacuolization.

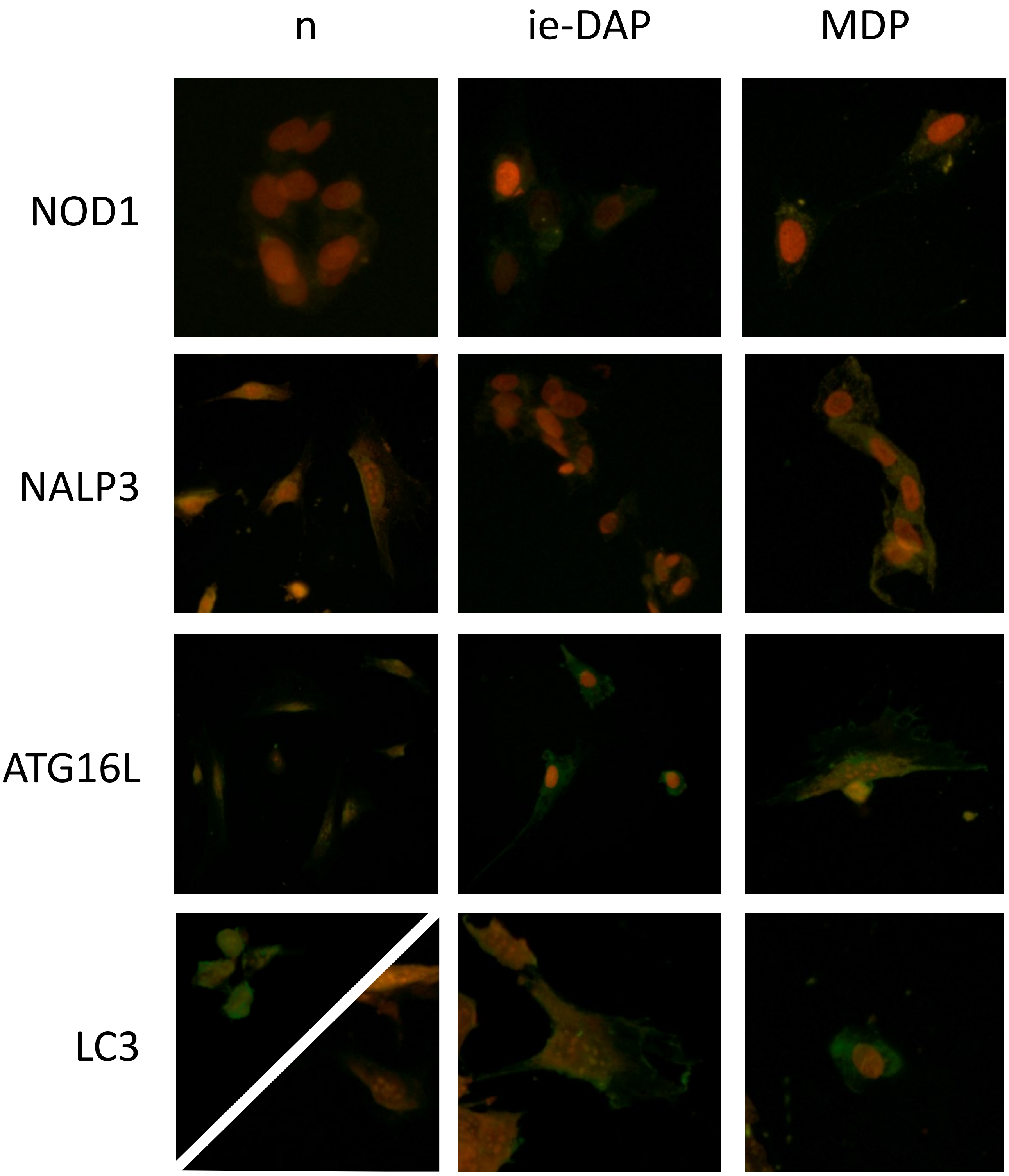

Supplemental Figure 2. Confocal fluorescence images of pre-pubertal primary Seroli cells isolated from 18 day BALB/c male mice, under intact (n) or challenged with either NOD1 (ie-DAP) or NOD2 (MDP) specific ligand. The cells were stained for NOD1, NALP3, ATG16L and LC3 using specific primary antibodies (specified in *Supplementary Table 2*) and secondary antibodies conjugated with FITC (●). Nuclei were stained using Propidium Iodide (●). The non-treated pre-pubertal Sertoli cells stained for LC3 are represented by two microphotographs.

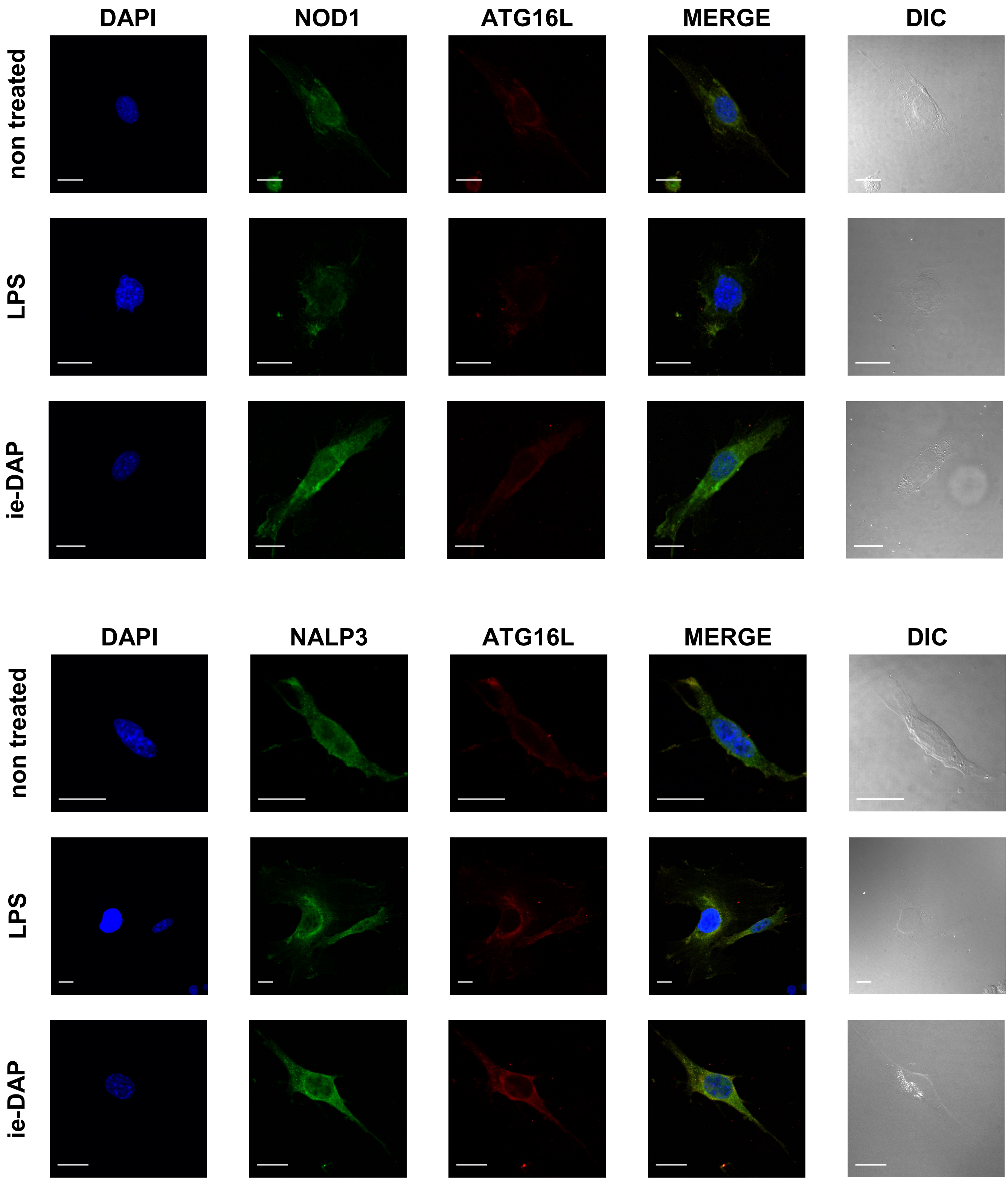

Supplemental Figure 3. Confocal fluorescence images of Sertoli cell line 15P-1 under intact or challenged with either LPS or ie-DAP conditions, stained for NOD1 (●), NALP3 (●) and co-stained for ATG16L (●).

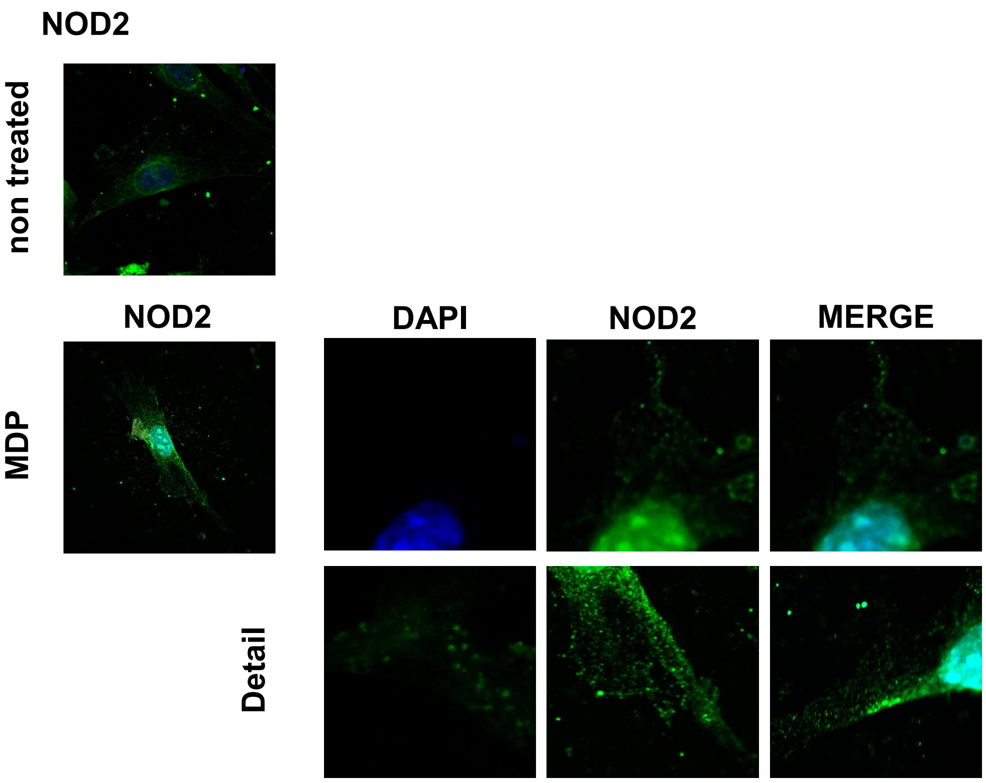

Supplemental Figure 4. Confocal fluorescence images of Sertoli cell line 15P-1 under intact or challenged with MDP conditions, stained for NOD2.

Detail section presents zoomed in portions of the cytoplasm with characteristic NOD2 positive stained granules.

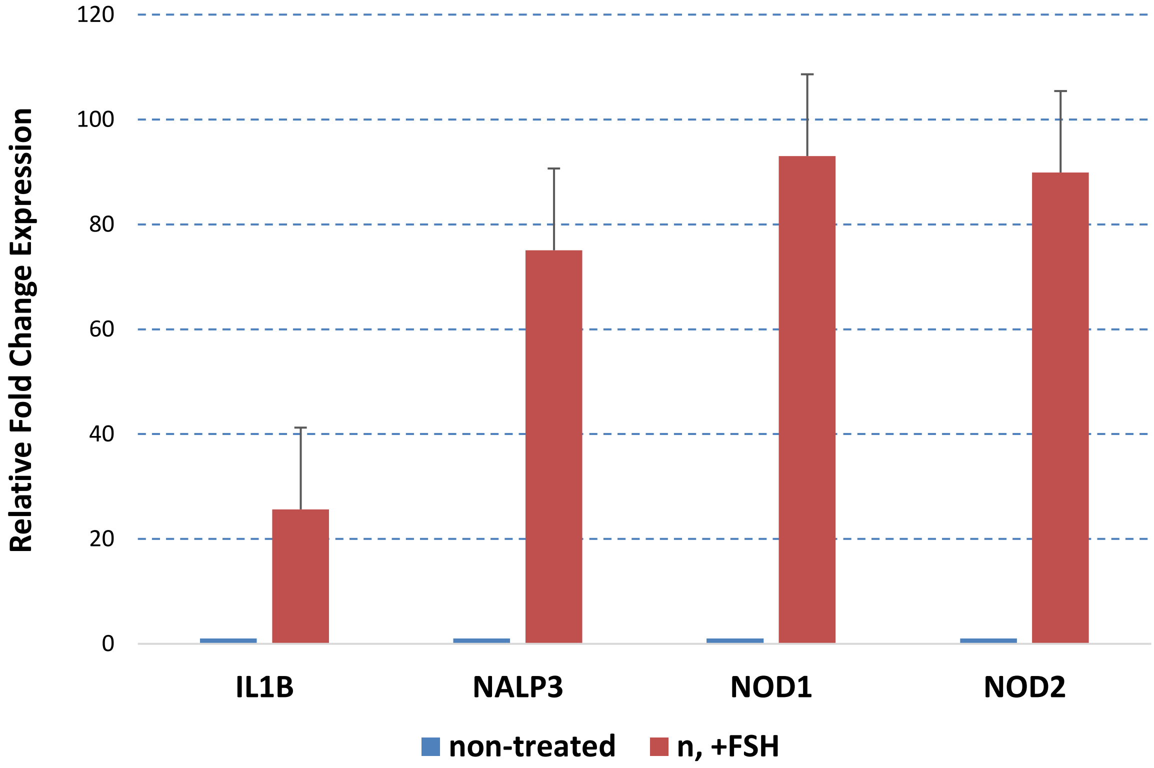

Supplemental Figure 5. FSH influences NOD1, NOD2 and NALP3 mRNA expression in primary Sertoli cells, 15d, grown in hormone supplemented media in iE-DAP and MDP challenged conditions

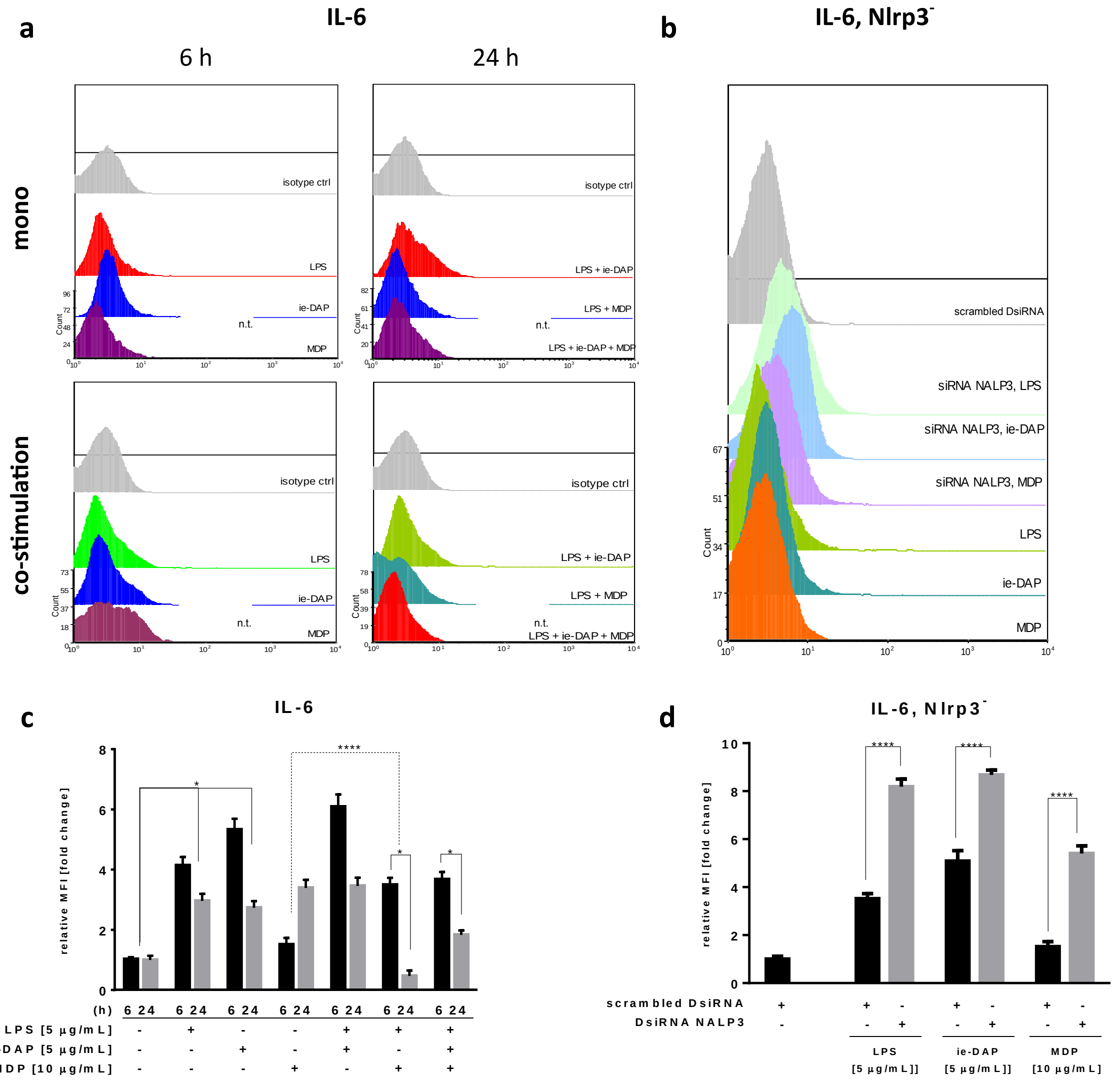

Supplemental Figure 6. Reciprocal regulation of IL-6 by TLR4/NOD1 and NOD2/NALP3 signaling

a, b. Intracellular cytokine flow cytometry assessment of IL-6 abundance in 15P-1 cells subject to challenge with LPS, ie-DAP and MDP for 6 h or 24 h. E. IL-6 flow cytometry assessment after DsiRNA NALP3 silencing (72h) followed by similar ligand challenge (6h). Gated and preliminary compensated cell events were acquired and represented as 3D Overlaid Histogram of individual experiment event counts towards measured channel log fluorescence. ***c, d.*** Expression is evaluated by the MFI indexes fold induction from the Histogram overlay analysis (**A, B**). Error bars indicate SD. Data are representative of three independent experiments with three technical replicates; ****, P < 0.0001; **, P < 0.005; *, P < 0.01

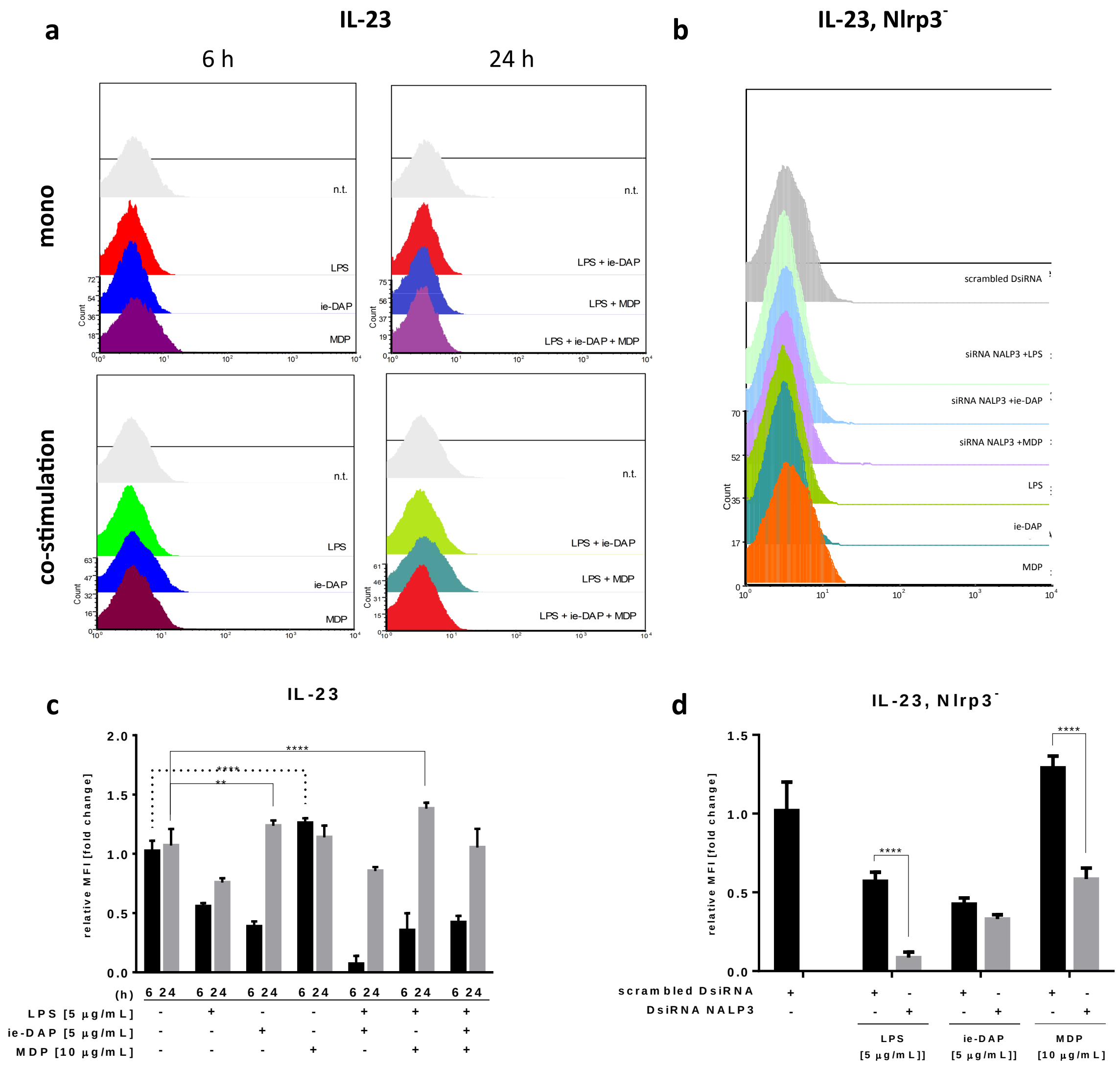

Supplemental Figure 7. TLR4 have suppressive, while NOD1 and especially NOD2 cross-talk most probably have inductive effect on the production of IL-23 by Sertoli cells

a, b. Intracellular cytokine flow cytometry assessment of IL-23 abundance in 15P-1 cells subject to challenge with LPS, ie-DAP and MDP for 6 h or 24 h. E. IL-23 flow cytometry assessment after DsiRNA NALP3 silencing (72h) followed by similar ligand challenge (6h). Gated and preliminary compensated cell events were acquired and represented as 3D Overlaid Histogram of individual experiment event counts towards measured channel log fluorescence. ***c, d.*** Expression is evaluated by the MFI indexes fold induction from the Histogram overlay analysis (A, B). Error bars indicate SD. Data are representative of three independent experiments with three technical replicates; ****, P < 0.0001; **, P < 0.005; *, P < 0.01

a

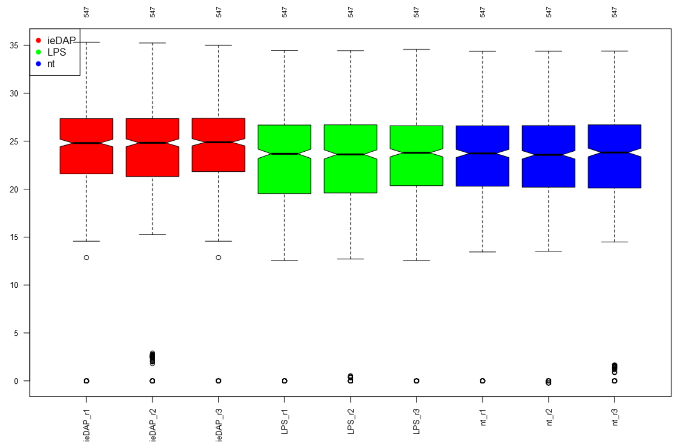

b

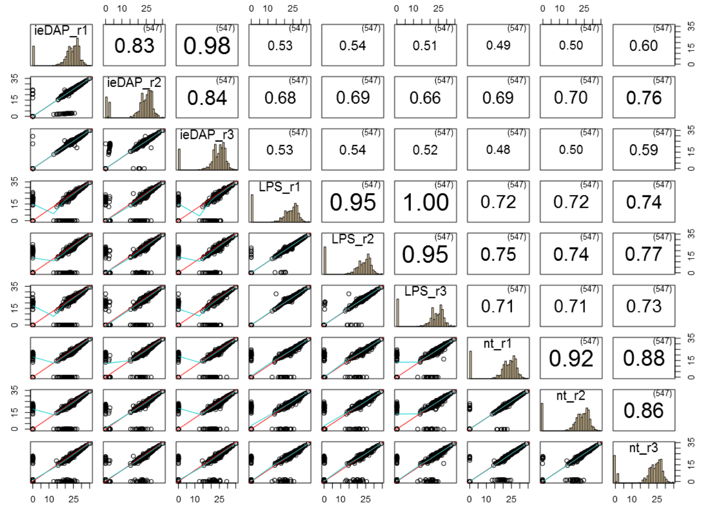

c

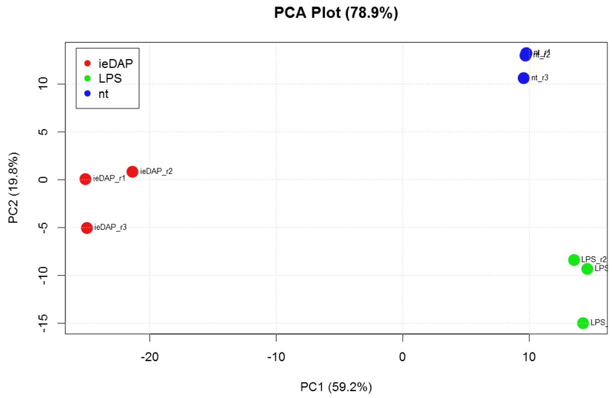

Supplemental Figure 8. Proteomics Data Pre-processing:

LTQ Orbitrap Proteomics data on differential protein expression of LPS/ie-DAP treated (24h) and control Sertoli cells were pre-processed using LOESS normalization of ANOVA filtered for significant difference, Log2 transformed data, with 0 values imputed with 1 (**a**). Correlation plot-scatter style of LOESS transformed data (**b**) and 2D projection of PC1 and PC2 of Principle Component Analysis of different treatment groups (**c**) are shown as well.

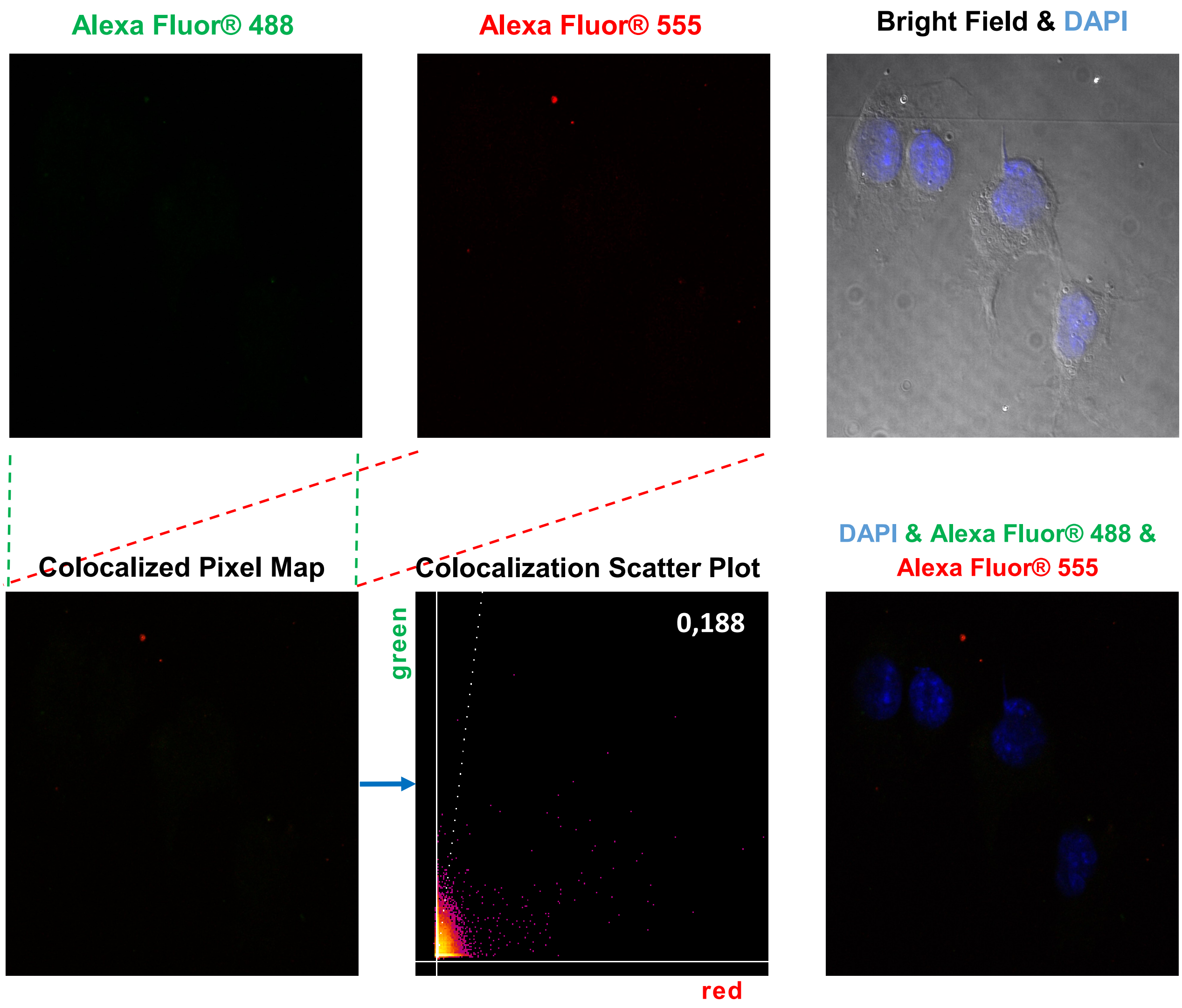

R_{total}	m	b	Ch1 thresh	Ch2 thresh	R_{coloc}	R < thresh	M1	M2	t_{M1}	t_{M2}	N_{coloc}
0,188	7,587	-607,0	87	53	0,2021	-0,006	10,000	10,000	10,000	0,9335	232821

Supplemental Figure 9. Validation of the Co-localization experiments:
Following secondary antibodies were used:

- Invitrogen anti-rabbit IgG conjugated with Alexa Fluor 488 (emitting in green spectrum)
- Invitrogen anti-goat IgG conjugated with Alexa Fluor 555 (emitting in red spectrum).

The images were acquired using Nikon NIS Elements AR software and transferred to Fiji (ImageJ implementation) module for co-localization assessment. R_{total} (intact acquired images) was estimated of 0.188

Negative controls of Sertoli cells stained only with the specified secondary antibodies did not show significant co-localization due to their emitting spectra and non-specific staining patterns.

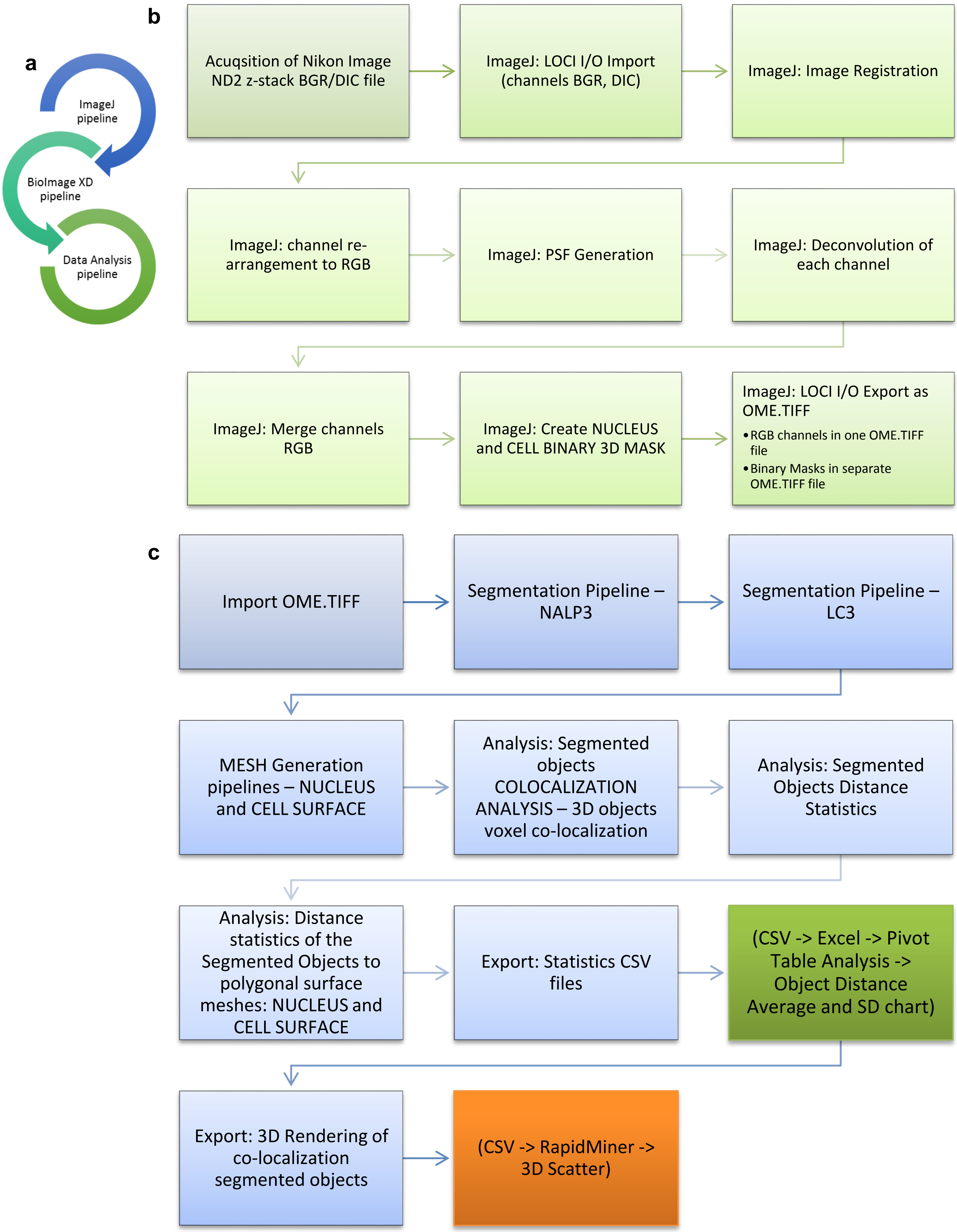

Supplemental Figure 10. Full workflow from Image acquisition to segmented objects statistical analysis. a. ImageJ image processing and data flow. **b., c.** BioImage XD flow and data export to RapidMiner data mining suite. The exported CSV files are processed with Excel (PivotTable analysis, estimated variable averages and standard deviations, generated Avg.Obj. Distance, Avg. Obj. Vol, Number of colocalized Obj., Percentage of colocalized Voxels, and distance bar charts. Same CSV imported in RapidMiner were used for generation of 3D scatter plots and Principle Component Analysis and Independent Component Analysis results and 3D scatters.

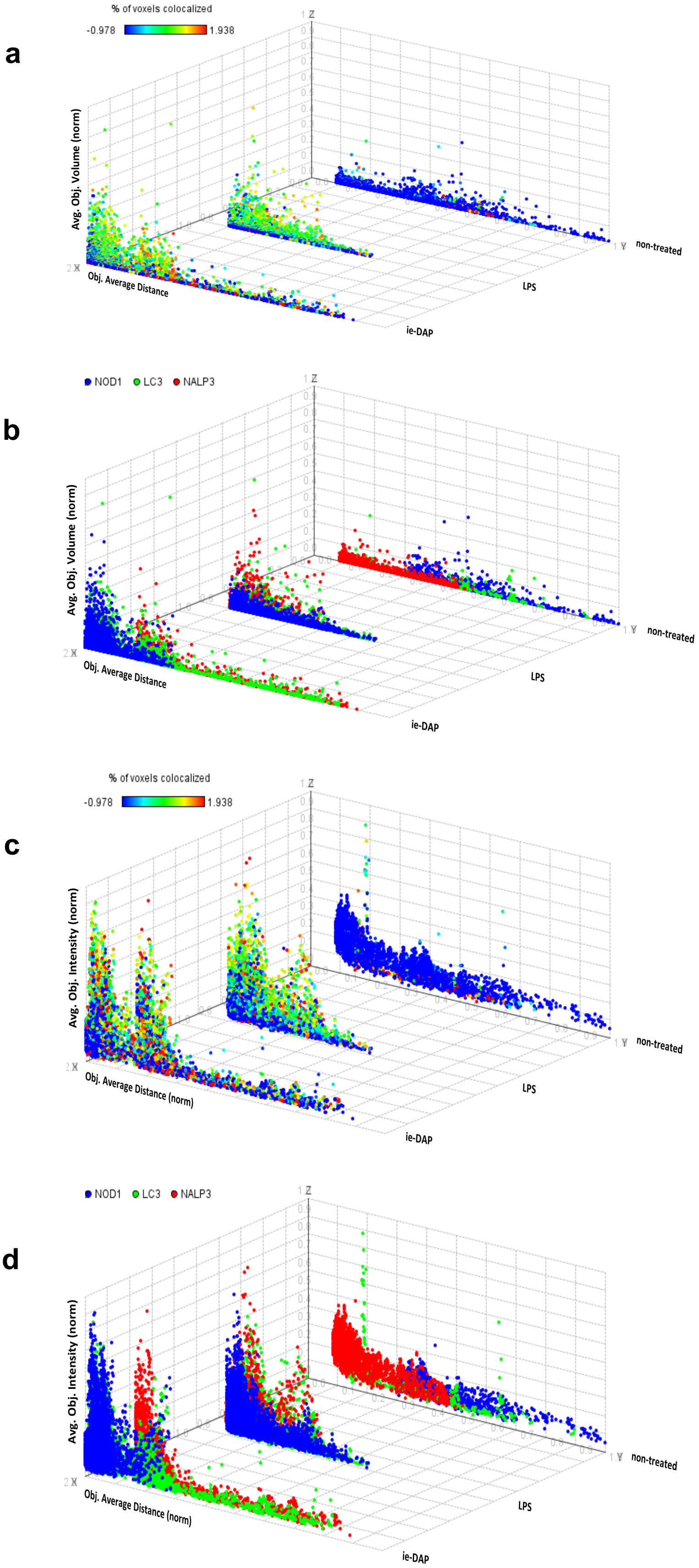

Supplemental Figure 11. Segmented objects distribution by Obj. Avg. Distance or Obj. Avg. Intensity (x) plotted against Obj. Avg. Volume and grouped by treatment modality, presented using 3D scatter plot. Dots are colored either based on voxel co-localization percentage (0-1) linear scale mapping or solid color based on object molecular class (NOD1, blue; LC3, green; NALP3, red).

a

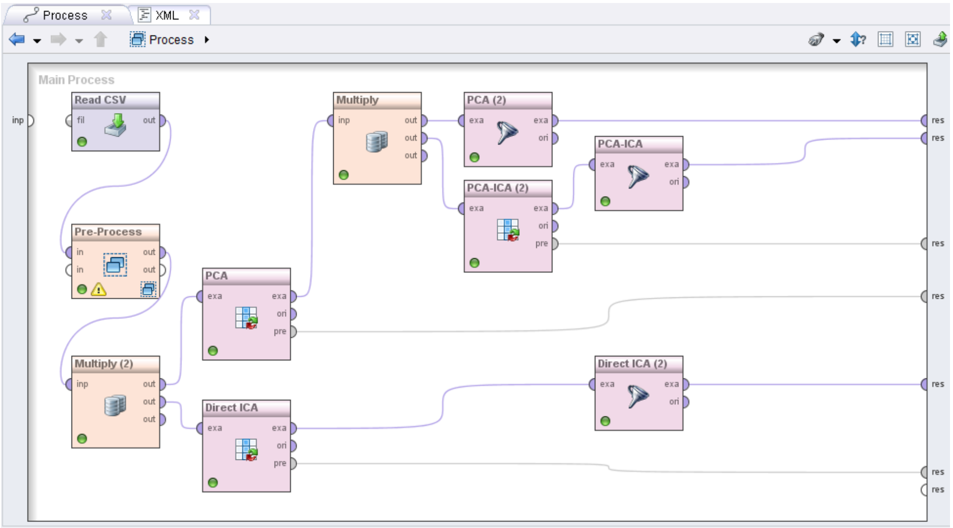

b

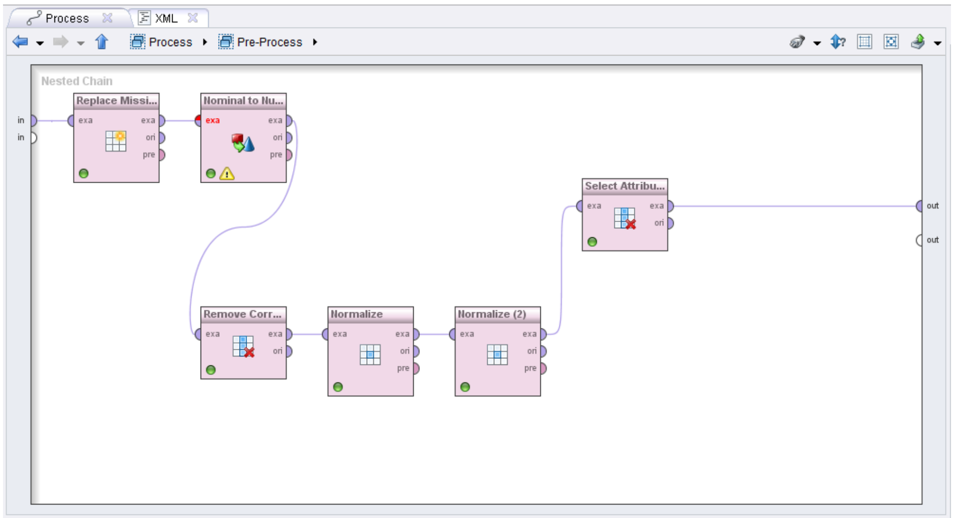

Supplemental Figure 12. a. RapidMiner data flow of the analyses performed (PCA and ICA) on the CSV files exported from BioImage XD containing segmented objects image data. **b. RapidMiner nested process flow for data preprocessing** — normalizations, missing value removal and attribute selection

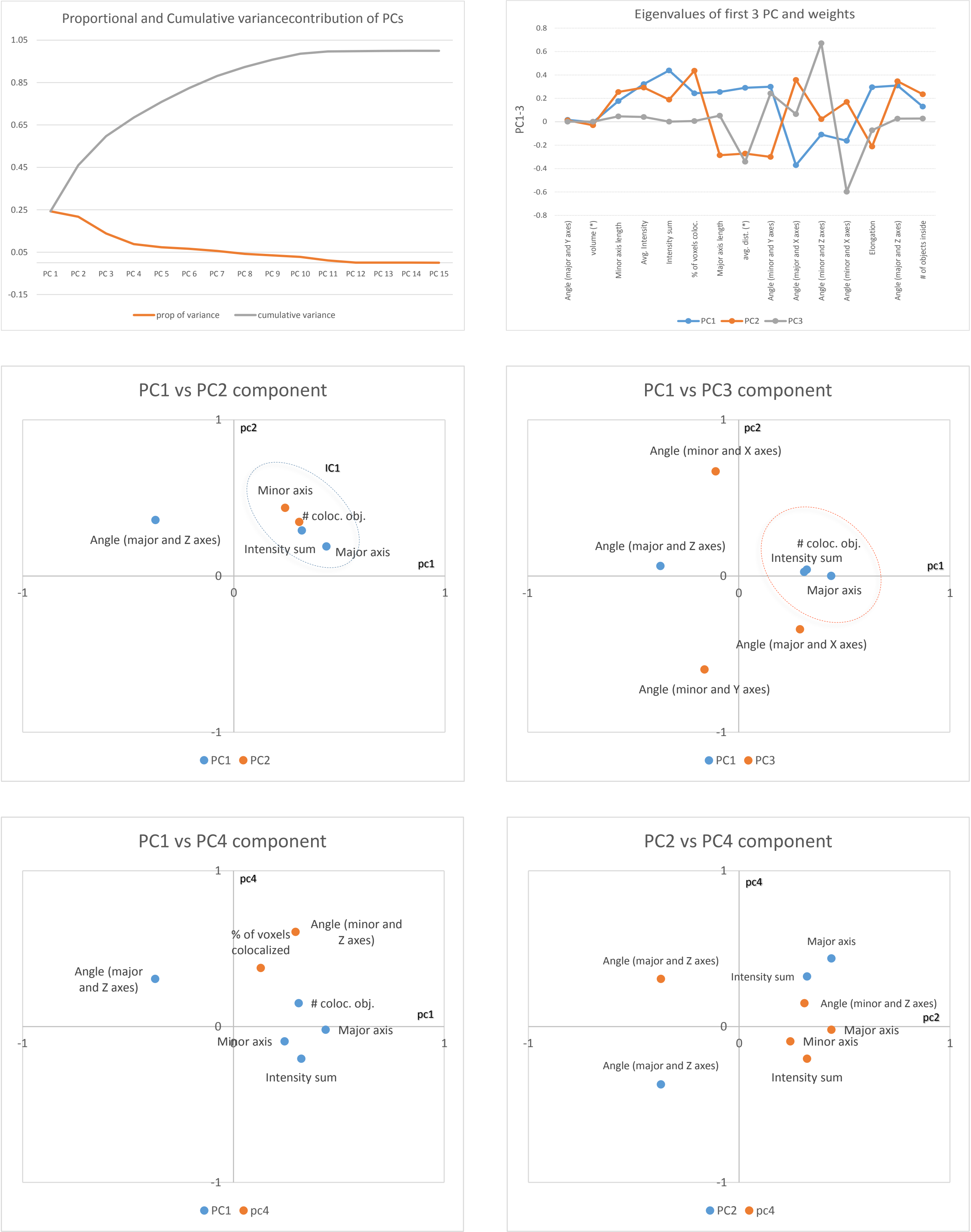

Supplemental Figure 13. Variance plot (scree) of the principle components showing those with highest impact on the total variance. Eigenvalues of highest variance impeding principle components PC1-PC3 are plotted against the real object attributes, showing those attributes that have highest influence on each principle component.

Several principle component 2D charts plotting each original attribute against selected principle components, with object attributes eigenvalues representing abscise and ordinate (x,y) values respectively.

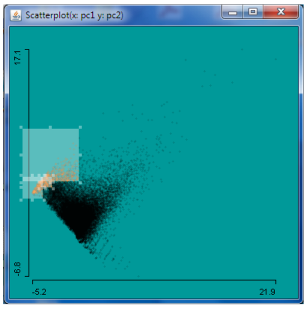

Selection of segmented data from cluster "Upper Left", belonging to objects having treatment attribute "iE-DAP".

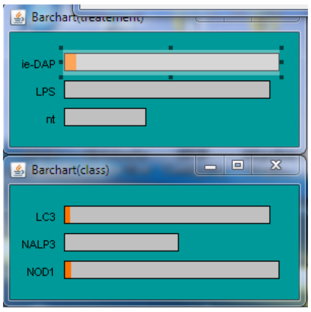

The logical clause "AND" upon selection is applied in the highlighted box on the upper window.

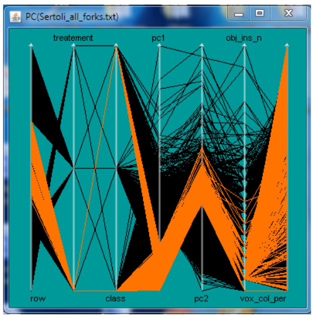

Cross-linked chart showing the values of some attributes of the selected objects.

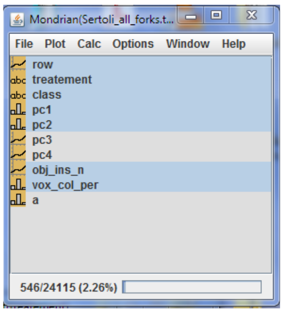

Selection of attributes to be exported for the selected objects (TAB delimited flat text file).

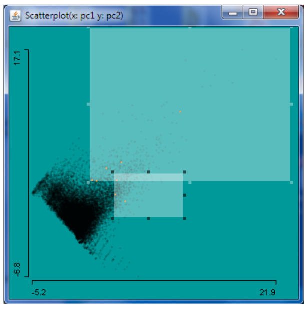

Few objects were found in cluster "Upper Right" to of the group of non-treated cells, but to belong to all of the three molecular types.

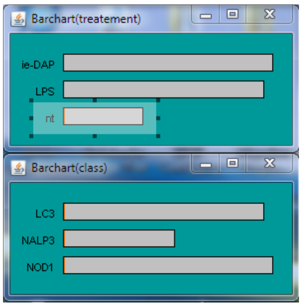

The objects from the selected cluster were filtered to belong only to treatment attr. "nt", i.e. non-treated

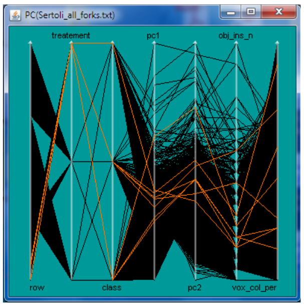

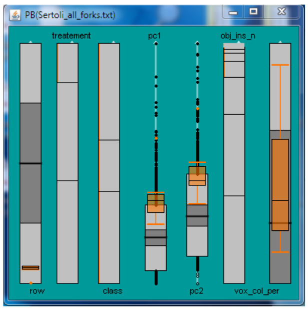

The cross-link variable chart and selected variable instances distribution plot demonstrated high number of object co-localization and wide distribution voxel level co-localization.

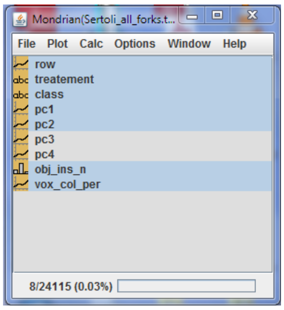

Selected attributes of the filtered objects were exported and analyzed in Excel, see Main Figure 4C.

Supplemental Figure 14. Algorithm of clustering single particle expression data after RapidMiner step.

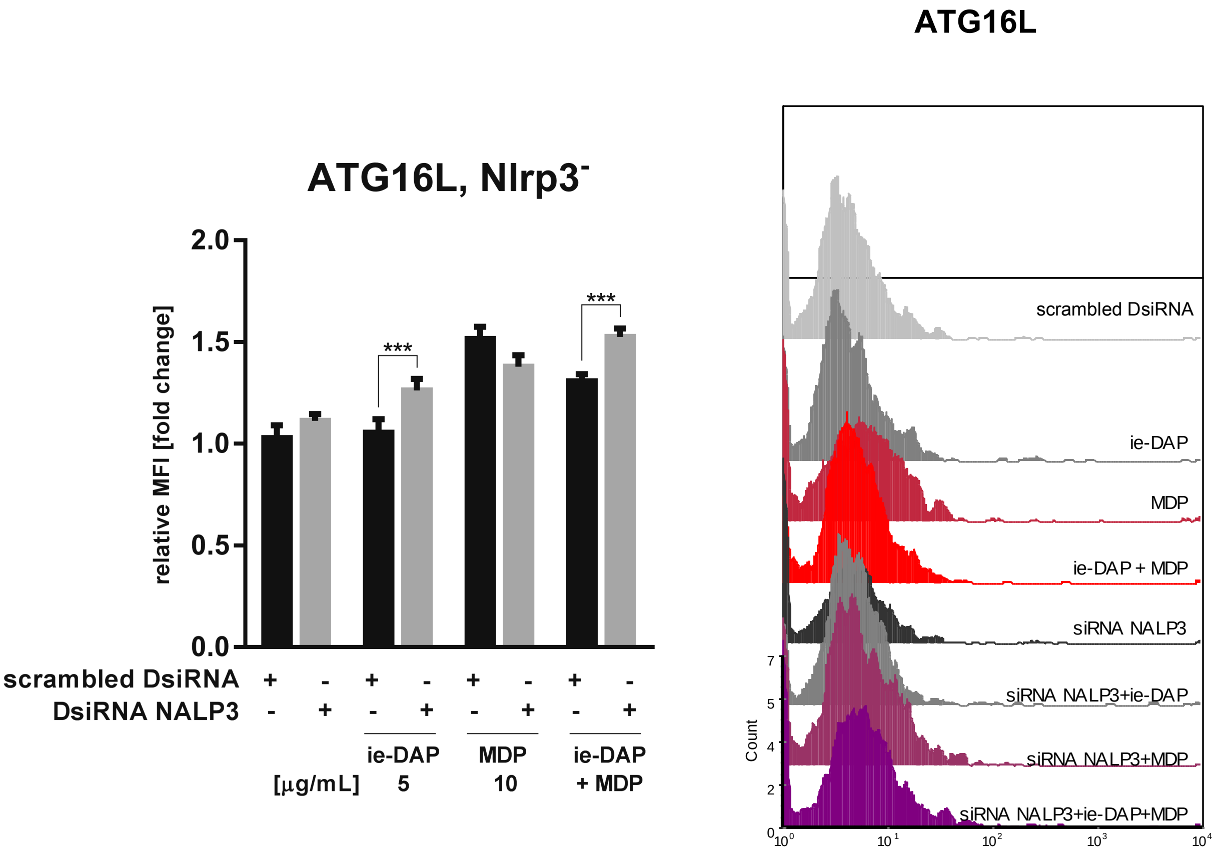

Supplemental Figure 15. Intracellular cytokine flow cytometry assessment of ATG16L abundance in 15P-1 cells subject to challenge with LPS, iE-DAP and MDP for 24h; DsiRNA Nlrp3 silencing (72h); Gated and preliminary compensated events were acquired and represented as 3D overlaid histogram *of* event counts *vs*. channel log fluorescence. Expression is evaluated by the MFI indexes fold induction from the histogram overlay analysis.
